# Olive Trees By-Products as Sources of Bioactive and Other Industrially Useful Compounds: A Systematic Review

**DOI:** 10.3390/molecules26165081

**Published:** 2021-08-22

**Authors:** Valentina Lo Giudice, Immacolata Faraone, Maria Roberta Bruno, Maria Ponticelli, Fabiana Labanca, Donatella Bisaccia, Carmine Massarelli, Luigi Milella, Luigi Todaro

**Affiliations:** 1 School of Agricultural, Forestry, Food and Environmental Sciences, University of Basilicata, Viale dell’Ateneo Lucano 10, 85100 Potenza, Italy; valentina.logiudice@unibas.it (V.L.G.); brunomroberta@gmail.com (M.R.B.); luigi.todaro@unibas.it (L.T.); 2 Department of Science, University of Basilicata, Viale dell’Ateneo Lucano 10, 85100 Potenza, Italy; immacolata.faraone@unibas.it (I.F.); maria.ponticelli@unibas.it (M.P.); fabiana.labanca@unibas.it (F.L.); 3 Spinoff BioActiPlant s.r.l., Department of Science, University of Basilicata, Viale dell’Ateneo Lucano 10, 85100 Potenza, Italy; 4 Italian National Research Council—Water Research Institute, Viale F. De Blasio 5, 70123 Bari, Italy; donatella.bisaccia@ba.irsa.cnr.it (D.B.); carmine.massarelli@ba.irsa.cnr.it (C.M.)

**Keywords:** specialized metabolites, bioactivity, circular economy

## Abstract

The need to produce an ever-increasing quantity of material products and food resulting from the planet globalization process has contributed to the spread of modern agriculture based on a linear production resulting in the generation of tons of waste. This huge amount of waste is generally accumulated in landfills, causing different environmental problems. Hence, researchers moved on to study the processes used to recover agro-industrial by-products within a circular and sustainable bio-economy concept. A systematic quest on Scopus and PubMed databases was performed to identify the data available to date on recycling agro-industrial by-products of *Olea europaea* L. This systematic review summarizes the knowledge regarding the use of olive trees by-products for producing animal feed, biocomposites, bioethanol, cellulose pulp, activated carbon, and as a fuel source for energy production. Furthermore, the data regarding the potential biological activity of extracts from olive roots, wood, bark, and pruning were analyzed. Olive trees by-products are, indeed, rich in molecules with antioxidant, antimicrobial, cardioprotective, and anticancer activity, representing a promising candidate for treat several human diseases.

## 1. Introduction

Agriculture has always been fundamental in providing material and food for humanity to survive. Initially, agriculture was founded on circular sustainability models based on using available resources and plants only to guarantee the local community’s survival. However, population growth and planet globalization have contributed to the spread of linear-producing modern agriculture to achieve the best production with maximum profit and lower cost. In particular, following the United Nations’ previsions [1], it is expected that the global population will increase from 7.7 billion in 2019 to 9.7 billion in 2050. This forecast leads to several concerns about the consumption of minerals, biomass, metals, and fossil fuels, which are expected to double the annual waste products as a consequent increase by 70% in the next 40 years [2]. The overproduced organic waste derived from agricultural and food industries is often not reused and ends up being accumulated, creating various environmental problems [3]. Hence, the need for by-products re-utilization to avoid or minimize their release in the environments has developed. From this necessity, the concept of circular economy (CE), based on two fundamental goals, reduction of energy consumption and waste re-utilization or management, was born. To stimulate and encourage these actions, the European Commission launched a first Circular Economy Action Plan in 2015, then implemented in 2020 with new legislative and non-legislative measures focusing on sustainable product design, empowering consumers and public buyers, and circularity in production processes [4]. In particular, at the basis of the CE there are the 6Rs (reuse, reduce, redesign, recycle, recover, and remanufacture) [5], which are aimed to transform waste materials into input for other industrial processes or in regenerative resources for nature (i.e., composite) [4]. To date, different uses of agro-industrial waste residues have been proposed, such as a source of energy (biogas and biofuels), fertilizers, animal feed, and bioactive compounds; the scientific community is paying close attention to their valorization. In this context, *Olea europaea* L. agro-industrial by-products could represent a natural source with consequent application in different fields. To encourage the use of plant wastes/by-products and implement the circular economy, this systematic review provides an overview of *O. europaea* agro-industrial by-products’ alternative uses.

*O. europaea* is a small evergreen and long-lived tree belonging to the family Oleaceae. It is thought that it is native to Mainor Asia [6] and now is considered one of the most important fruit trees of the Mediterranean basin. Following the Food and Agricultural Organization of the United Nations, 2.7 million tons of olive oil was manufactured annually around the world, with Italy (23.1%), Spain (35.2%), and Greece (16.1%) as the leading producers [7]. For years, the olive tree’s chemical composition has been investigated, and it was seen that phenolic compounds constituting an interesting group of molecules are found in olive fruits, leaves, wood, bark, roots, and stones [8]. However, only recently olive trees agro-industrial by-products have been paid attention to, as olive tree cultivation and olive oil production generate a significant amount of biomass, mostly from pruning. This compels to think that in Italy, from older olive trees, depending on the trees’ structure and the size, an average of between 10 and 30 kg of wastes was produced. The period in which residues are available is from January to April; operations are carried out at different intervals, from once a year to once every 3–4 years, depending on the variety, the environmental parameters, and the level of plantation specialization. The average production of olive pruning residues is therefore about 1.7 tons/ha; considering that approximately 1,100,000 hectares are used as olive groves in Italy, theoretically, it would be possible to obtain about 1,870,000 tons of biomass [9]. Olive oil by-products are formed mainly by wood, which is generally underutilized since it is primarily operated for home heating. Thus, several studies have been devoted to searching for more profitable uses. From these feedstocks are produced animal feed, biocomposites, bioethanol [10], cellulose pulp [11], activated carbon [12], and as a fuel source for energy production [13]. All these are made possible owing to the “biorefinery” approach, which uses different technologies that can isolate the components of biomass into basic elements (proteins, carbohydrates, fats, etc.), which can then be transformed into value-added chemicals, biofuels, and other products. Separation generally occurs in a single implant or in a network of implants that integrate biomass conversion processes and equipment to produce biofuels, chemicals, and energy [14]. Noteworthy is the recovery of bioactive compounds from olive trees by-products. Indeed, phenolic compounds recovery can be achieved by obtaining products that can be reintroduced into the economy as new raw material, i.e., for the production of nutraceutical products or feed and food additives. Phenolic compounds are an essential group of secondary metabolites that plants produce in response to stimuli derived from the environment. Generally, these molecules’ amount depends on the target species, on the location, and several biotic factors like bacteria, herbivores, etc. [15]. In particular, phenolic compounds are synthesized due to plants’ necessity to survive, so they possess a protective function related to their antioxidant and antimicrobial properties [16]. Further, they have healthy properties since several reports demonstrated their anti-proliferative, anticancer, anti-inflammatory, and anti-obesity activity. As previously said, phenolic compounds are abundant in olive trees and in the last year, particular attention has been paid to the recovery of these compounds from olive wood, bark, pruning, and roots. The first section of this review summarizes the data focused on the antioxidant, antimicrobial, cardioprotective, and anticancer activity of extract from olive trees by-products, while the second section is centred on olive tree waste industrial bioconversion.

## 2. Results and Discussion

### 2.1. Study Analysis

**Figure 1:**
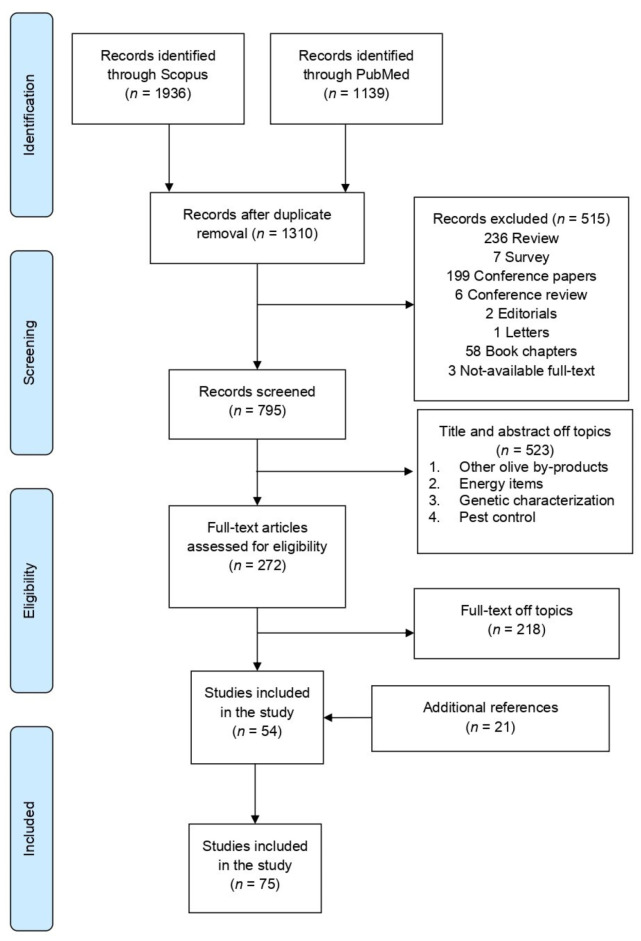
Flow diagram of the systematic review literature search results based on PRISMA statement.

A preliminary survey of literature led to the identification of 3075 reports (1936 from Scopus and 1139 from PubMed). After checking duplicates, 1765 results were removed and considered only once, resulting in 1310 articles. A primary screening based on title and abstract resulted in excluding 523 manuscripts that did not meet the inclusion criteria or were considered off-topic. Finally, 272 articles were fully examined and, out of those, 218 were excluded. Besides, extra 21 suitable articles were included after analyzing the reference list of the selected literature by title, abstract, and full-text. As described by the flowchart of Figure 1, the selection process of the bibliographic sources led to the final selection of 75 papers for data extraction.

**Figure 2:**
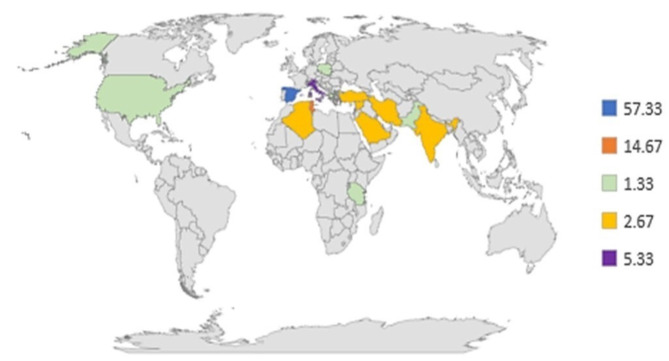
Distribution of articles by country. Spain, 57.33%; Tunisia, 14.67%; Italy, 5.33%; India, Iran, Arabia, Algeria, Turkey, and Syria, 2.67%; Greece, Pakistan, Tanzania, USA, Poland, 1.33%.

Among those, the majority have been published in Spain (57.33%), followed by Tunisia (14.67%), Italy (5.33%), and others (Figure 2). This distribution should be explained by the optimal growing conditions of olive trees in those areas, leading to the availability of a significant amount of olive by-products for analysis. The map also highlights that the origin of the papers coincides with the greatest oil world producers.

**Figure 3:**
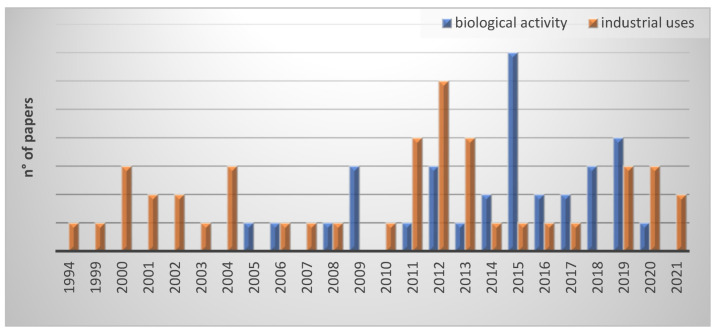
Distribution of studies by year of publication, focusing on industrial uses (orange) and biological activity (blue).

In this review, the articles included were also analyzed by year of publication, underlining that they remark the slow birth of the Green Chemistry approach during the 1990s. Indeed, the valorization of by-products is one of the key points, as shown in Figure 3. The increased number of published articles regarding by-product uses can be observed since 1994, primarily concerning industrial applications and then biological uses.

**Figure 4:**
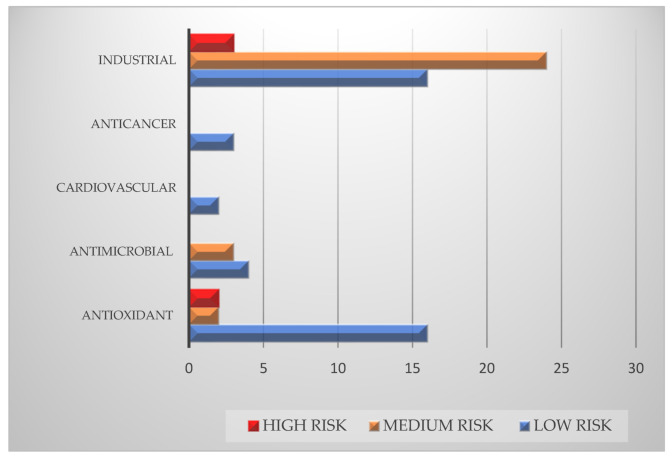
The assessment of bias risk is based on a checklist adapted from the Cochrane Handbook for Systematic Reviews of Interventions [17] and Collaboration for Environmental Evidence (CEE) Guidelines and Standards for Evidence Synthesis in Environmental Management, in conformance to ROSES reporting standards [18,19]. The studies regarding the different biological activities and the industrial uses have been classified into high (red), medium (orange), and low (blue) risks of bias.

The assessment of bias risk, based on a checklist adapted from the Cochrane Handbook for Systematic Reviews of Interventions [17] and Collaboration for Environmental Evidence (CEE) Guidelines and Standards for Evidence Synthesis in Environmental Management, in conformance to ROSES reporting standards [18,19] isreported in the bar graph of Figure 4. The majority of included articles fall into the low-risk of bias category, enhancing the overall quality of the manuscript.

### 2.2. Chemistry O. europaea

The olive tree is one of the most studied plants, and numerous studies concerning its phytochemical characterization have revealed the presence of numerous bioactive secondary metabolites belonging to different chemical classes (Table 1).

In general, the main phenolic compounds found in *O. europaea* are oleuropein, hydroxytyrosol, and tyrosol. These compounds have important antioxidant activities and they are present in the roots, albeit in low concentrations compared to other parts of the plant [20].

Furthermore, in olive tree roots and rhizospheric soil, there are mannitol, sorbitol, and myo-inositol [21]. Interesting is the influence of arbuscular mycorrhizal fungi colonization on the content of flavonoids, phenolic compounds, and soluble carbohydrates in olive tree roots. In fact, mycorrhizal plants showed a higher content of flavonoids and total phenols with enhanced levels of sucrose and fructose in mycorrhizal roots. Instead, glucose amounts stayed constant [22].

The olive bark of the “*Chemlali*” cultivar was characterized by an important concentration of oleuropein followed by a poor concentration of coumarin and vanillic acids. In general, oleuropein is the main glycoside in the olive tree. This compound is responsible for the bitter taste of olive products and it is known mainly as a strong antioxidant and for its human health benefits [23]. 

In 2015, Tóth et al. identified 41 different compounds in the methanolic extracts of olive barks (5 hydroxycoumarins, 16 secoiridoids, 8 flavonoids, 9 LMWPs, and 3 cinnamic acid derivatives). The identified hydroxycoumarin derivatives were present in the barks but not in leaves. They are dimeresculetin, esculetin, esculin, cichoriin, or an isomer of esculin, and methylesculetin, a compound that differs from esculetin only in a methyl group. In general, the olive tree is rich in secoiridoids, and in particular, in oleosides, which are typical of the Oleaceae family. Among all, barks contained, mainly in the periderm, oleuropein and three oleuropein isomers. Other identified secoiridoids in periderm were oleoside and secologanoside. Moreover, the authors showed that rhytidome is rich in the flavonoid naringenin and the cinnamic acid derivative acteoside and acteoside isomers [24].

The woody portion of olive trees is a source of natural bioactive compounds of potential interest for the food industry. For this reason, the wood has been extensively studied. For example, wood Tar oil extracted with ether and its main constituents were acetic acid, acetaldehyde, colchifoleine, d-cycloserine, malonamic acid, and kaempferol. Colchifoleine is known for its antioxidant and antibacterial activities. Acetic acid, instead, is used as a bactericidal agent [25]. 

Pérez-Bonilla et al. extracted a wood sample of *O. europaea* with solvents of increasing polarity (hexane, dichloromethane, ethyl acetate, and 96% ethanol). The ethyl acetate extract was subjected to phytochemical characterization, resulting in tyrosol, hydroxytyrosol, cycloolivil, 7-deoxyloganic acid, ligustroside, and oleuropein [26]. 

Another wood sample collected from the pruning of a tree placed in Spain was extracted by maceration with dichloromethane and then with ethyl acetate. Then the study led to the isolation and identification of the antioxidants present in ethyl acetate extract. Seven secoiridoids were isolated, oleuropein-3″-methyl ether, 7″*S*-hydroxyoleuropein, jaspolyanoside, ligustroside 3′-*O*-*β*-d-glucoside, jaspolyoside, isojaspolyoside A, and oleuropein 3′-*O*-*β*-d-glucoside [27]. 

The same authors in the next study selected, among fifty olive wood extracts, an ethyl acetate sample and detected secoiridoids ligustroside and oleuropeinas the main components. Moreover, they make an acid hydrolysis pretreatment of olive wood samples before the extractions, but the experiment did not improve the results. Instead, from the ethanolic extracts of olive wood, they identified the compounds (7″*R*)-7″-ethoxyoleuropein and (7″*S*)-7″-ethoxyoleuropein [28].

To acquire knowledge on the chemical composition of olive tree wood, the authors thoroughly studied an ethyl acetate extract and obtained two new monoterpene glycosides, (−)-oleuropeic acid 6′-*O*-α-d-glucopyranosyl ester and (−)-perillic acid 1′-*O*-*β*-d-primeverosyl ester, together with (−)-olivil, (−)-olivil 4-*O*-*β*-d-glucopyranoside, (−)-oleuropeic acid, (−)-oleuropeic acid 1′-*O*-*β*-d-glucopyranosyl ester, (−)-oleuropeic acid 6′-*O*-*β*-d-glucopyranosyl ester, the aldehydic form of oleuropein aglycone and (+)-1-hydroxypinoresinol 1-*O*-*β*-d-glucopyranoside [8]. 

When the wood samples are extracted using methanol in Soxhlet apparatus, besides hydroxytyrosol, tyrosol, and oleuropein, vanillin, protocatechic acid, *p*-coumaric acid, and benzoic acid were also identified [29,30].

The wood of ten main Spanish olive cultivars was collected during the pruning works and extracted with dichloromethane and ethyl acetate at reflux under nitrogen atmosphere. The ethyl acetate extracts were subjected to phytochemical characterization identifying 10 secoiridoids, 3 lignans, 2 phenol alcohols, 1 iridoid, and 1 flavonoid. Qualitative and quantitative differences were present among olive cultivars. The major compound in all olive cultivars, except in cultivars Picual and Farga was the lignan (+)-1-hydroxypinoresinol 1-*O*-*β*-d-glucopyranoside [31].

Olive wood obtained from the pruning of cultivar Chemlali growing in Tunisia was extracted using methanol: water 80:20 (*v/v*) identifying several compounds classified as sugars, organic acids, phenolic aldehyde, simple phenolic acids, simple phenylethanoids, flavonoids, coumarins, caffeoyl phenylethanoid derivatives, iridoids, secoiridoids, and lignans. In particular, saccharides, organic acids, and iridoids are non-phenolic compounds. Among all, the most prevalent monosaccharides are d-mannitol, stachyose, and verbascose. The identified iridoids were loganic acid, loganin, and 7-deoxyloganic acid, and their glycosidic derivatives. The phenolic compounds identified in the study were vanillin and simple phenolic acids (not linked to phenylethanoids) or phenylethanoids (not linked to monoterpenes). Gallic, chlorogenic acid *p*-hydroxybenzoic acid, and caffeic acid, dihydroxybenzoic acid hexosidepentoside dihydroxybenzoic acid hexoside, and decaffeoylverbascoside were also identified. Among coumarins, there are scopoletin, aesculin, and its aglycone aesculetin. Instead, no flavonoids were identified in the work in olive wood. Instead, it contains a large number of secoiridoids between free forms, such as oleoside and elenolic acid derivatives (three isomers of 1-*β*-d-glucopyranosyl acyclodihydroelenolic acid), and phenol-conjugated secoiridoids such as oleuropein derivatives (oleuropein aglycone, oleuropein isomers, demethyloleuropein, oleuropein hexosides, hydroxyoleuropein, methoxyoleuropein, and dihydro-oleuropein). Finally, the identified lignans were olivil 4-*O*-*β*-d-glucopyranoside, cycloolivil glycoside, cycloolivil, olivil, and (+)-1-hydroxypinoresinol 1-*O*-*β*-d-glucopyranoside [32].

Usually, the olive tree wood trimmings contain 62.4% holocellulose, 36.3% *α*-cellulose, and 19.1% lignin by dry matter weight [33]. For these reasons, the olive tree wood was often submitted to pretreatment at 190, 210, 230, and 240 °C to improve enzymatic hydrolysis yields. In this study, the water-insoluble fiber was delignified by an alkaline peroxide treatment. In this way, it is possible to solubilize up to 80% of the lignin in the original wood, leaving a cellulose-rich residue with a concentrated glucose solution [34].

The characterization of natural fibers obtained from leaf, small branches, and large branches of the olive trees confirmed the different compositions of the other parts of the plant. In particular, the large olive branches present a relatively higher cellulose content (39.42% in total) compared to the fibers extracted from small olive branches and olive leaves. Moreover, it is possible to distinguish hemicellulose (24.23%) and lignin (14%) in cellulose content. These fibers can be utilized as natural reinforcement materials in the manufacturing for lightweight industrial applications [35].

The study on olive tree pruning residues is also interesting. In fact, olive pruning residues occur mainly during cultivation and are usually disposed of by open-air combustion directly in the field, which is also a possible source of pollution.

Dried olive pruning residue showed 40.2 wt% cellulose and 28.1 wt% lignin content. Bartoli et al. collected the bio-oils obtained from pruning olive trees and analyzed them to evaluate their composition and physico-chemical properties. These oils are dark-brown liquid with a low viscosity. The most abundant compounds identified in bio-oils of olive pruning residue were acetic acid, 1-hydroxy-2-propanone, furfural, 1,4-anhydro-d-mannitol. For these reasons, olive pruning residue can be a source for obtaining useful chemicals and fuels and represents a good possibility for reducing all of the environmental risks involved in their disposal [36].

Moreover, the separation of olive pruning into primary (stems > 1 cm diameter) and residual (stems < 1 cm diameter, and leaves) was carried out in a biorefinery. It was seen that the biomass is rich in hemicellulose decomposition products, cellulose, and lignin. In general, the composition of olive pruning analyzed is cellulose (glucan), hemicelluloses (xylan, arabinan, and acetyl groups), lignin, extractives, ash, carbon, hydrogen, nitrogen, and sulfur [37,38]. 

Mateo et al. have analyzed the composition of lignin, moisture, hemicellulose, cellulose, and ash of the olive tree pruning. This biomass consisted of branches, leaves, and pieces of trunks from olive trees collected during the pruning season. In this study, a pretreatment was used, and the percentage of lignin recovered was close to 100% [39].

Toledano et al. subjected the olive tree pruning to treatment for lignin extraction. This treatment consists of the biomass digestion in a mixture of ethanol:water (70 wt%) at 200 °C for 90 min in a pressure reactor. The result of syringol and guaiacol dealkylation, demethoxylation, and demethylation reactions was the highest for phenolic compounds (phenol, *o*-cresol, *m*-cresol, *p*-cresol, catechol, and 4-methylcatechol). Instead, from rough lignin alkaline nitrobenzene oxidation, the phenolic acids and aldehydes compounds vanillin acid, arabinose, syringic acid, vanillin, syringaldehyde, acetovanillone, and ferulic acid were released [40].

The autohydrolysis of olive tree pruning biomass can be useful forthe production of monosaccharides (glucose, xylose, galactose, arabinose, fructose), oligosaccharides (GlcOS, XOS, GalOS, MOS, AOS), and by-products (mannitol, AcO, acetic acid, formic acid, HMF, furfural). The major products obtained by autohydrolysis are xylooligosaccharides and glucooligosaccharides [41].

Olive tree pruning residues were also treated in a high-pressure reactor with a solution ethanol:water 60:40 (*v/v*). The analysis of monomeric sugars and hemicelluloses degradation by-products concentration in the original and sonicated organosolv black liquor identifies glucose, xylose, arabinose, acetic acid, formic acid, citric acid, lactic acid, xylitol. Moreover, the application of ultrasounds on organosolv liquor showed that degradation and/or decomposition processes could be favored by high temperature and high-pressure areas generated during sonication. This study demonstrated that the ultrasound treatment can intensify the biorefinery processes [42].

Conde et al. collected thin branches (<5 cm diameter) and leaves and identified in these residues principally oleuropein, hydroxytyrosol, tyrosol, homovanillyl alcohol, 3,4-dihydroxybenzaldehyde, vanillic acid, vanillin, and syringaldehyde. The majority of compounds were hydroxytyrosol, homovanillyl alcohol, and oleuropein [43]. 

In another study, olive tree biomass was collected in Spain after the fruit harvest. The sample was taken from branches cut from the tree and consisted of a mixture of leaves and wood from branches with different thicknesses. The chemical composition of samples reported by authors was glucose, phenolics, cellulose, hemicellulose, xylan, galactan, arabinan, mannan, acid-insoluble lignin, acid-soluble lignin, ash, and proteins. Regarding structural polysaccharides, a relatively high amount of cellulose (23.6%) and hemicellulose (15.6%) was found. Another structural component present is lignin (20.1%). The compounds identified are flavonoids such as taxifolin, rutin, luteolin, and its derivatives (mono and di-glucoside), chrysoeriol derivatives (glucoside), and apigenin derivatives (rutinoside), iridoid as loganin and derivative of loganic acid glucoside, lignan as (−)-olivil 4-*O*-*β*-d-glucopyranoside and secoiridoid hydroxyl oleuropein. Instead, the volatile compounds generated during pyrolysis were identified as benzaldehyde, *p*-vinylguaiacol, oguaiacol, and long-chain fatty acids (palmitic acid and stearic acids). The compounds furan 2,5-dimethyl, ethanone, 1-(2-furanyl)-, phenol, *p*-creosol, catechol, furfuralwere present in minor amounts [44].

Finally, in addition to oleuropein, Leila et al. demonstrated the presence of 2,4-di-tert-butylphenol (phenol,2,4-bis(1,1-dimethylethyl, 2,4-DTBP) which showed an anti CVB-3 activity [45].

### 2.3. Agro-Industrial By-Products from O. europaea and Their Biological Activity

**Figure 5:**
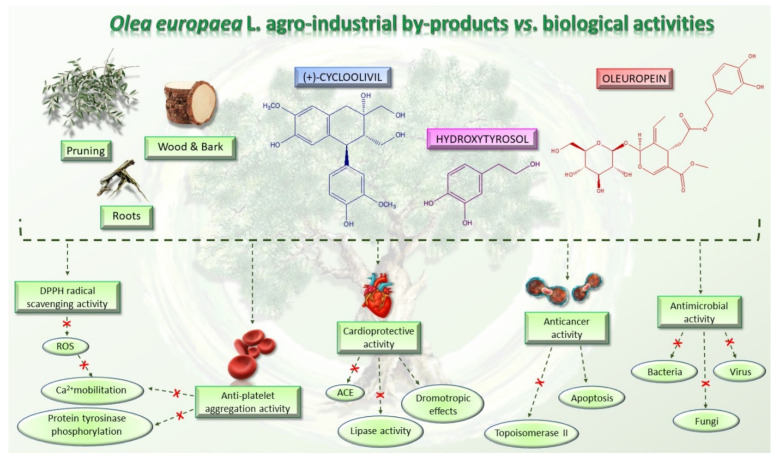
Graphical representation of *O. europaea* agro-industrial by-product biological activity. Extracts from olive roots, pruning, wood, and bark, showed antioxidant, antimicrobial, anti-platelet aggregation, and anticancer activities. These activities are related to the high content in bioactive compounds like oleuropein, hydroxytyrosol, and (+)-cycloolivil.

*O. europaea* has been investigated for a long time for treating microbial infection, obesity, inflammation, cardiovascular diseases, and cancer. Olive leaves, fruits, seeds, and oil are indeed used in traditional medicine alone or in combination with other drugs to treat several illnesses, and many scientific reports have confirmed their beneficial effects. However, the health benefits of olive trees’ agro-industrial by-products have been poorly studied. This section summarizes the knowledge reported to date on the potential biological activity of olive roots, wood, bark, and pruning (Figure 5).

### 2.4. Agro-Industrial By-Products from O. europaea and Their Antioxidant Activity

Oxidative stress has been implicated in various pathological conditions involving cardiovascular disease, cancer, neurological disorders (Alzheimer’s disease and Parkinson’s disease), diabetes, ischemia/reperfusion, and other ailments [46]. Reactive oxygen species (ROS) are also the main ones responsible for the complete degradation of lipid-containing food [47]. Furthermore, in recent years, some studies have been focused on the side effects linked to synthetic antioxidants widely used in the food industry, such as butylated hydroxyanisole (BHA), *tert*-butylhydeoquinone (TBHQ), and butylated hydroxy-toluene (BHT) [48]. For these reasons, much research is underway to discover new vegetables or agro-industrial by-products useable as a source of natural and safe antioxidants. In a particular way, the high availability and low cost of industrial and agricultural vegetable waste make them an interesting natural antioxidant source.

It is known that olives are rich in antioxidants responsible for the antioxidant activities ascribed to virgin olive oil. However, it is worth noting that the entire olive tree is an essential source of natural and safe antioxidants owing to the high amount of phenolic compounds that have been found in olive stems, leaves, wood, bark, roots, and stones [8,20,49,50,51]. In this review, special attention was paid to the antioxidant profile of wood, bark, and roots. In fact, owing to the high biomass amount annually generated by olive trees, pruning, olive wood, and bark are important agricultural by-products. These resources have been extensively investigated as a raw material for producing lignin, activated carbon, cellulose pulp, and bioethanol, but only recently, their chemical profile and the related antioxidant activity have been paid attention to. For antioxidants molecules isolated from plant material, solvent extraction is generally used and it has been found that extracts’ antioxidant activity is strongly related to the solvent used [52]. The most widely used test for determining extracts’ antioxidant activity is the 2, 2-diphenyl-1-picryl-hydrazyl-hydrate (DPPH) test since it is rapid, simple, and the radical is commercially available and stable. Besides, DPPH scavenging reaction has been suggested for measuring radicals’ rapid reactions, leading to the prediction of lipid oxidation. It was seen that extraction with dichloromethane permits removing non-polar components from olive wood with low antioxidant activity [53]. Contrarily, the better solvent for extracting antioxidants seems to be ethyl acetate or ethanol, where the first appeared to be more selective in extracting antioxidants from olive wood [26,27,28,54]. In particular, it was evidenced that although the yields of ethanolic extracts were higher than those obtained using ethyl acetate as a solvent, the most active extracts in terms of antioxidant activity were obtained using ethyl acetate by either direct extraction or sequential liquid-liquid partitioning procedures [28]. From these extracts, the main constituents responsible for the antioxidant activity of olive wood were subsequently isolated. Oleuropein and ligustroside are the secoiridoids found in high concentrations in the olive wood extract. Other compounds are the lignan (+)-cycloolivil, the phenolic alcohol hydroxytyrosol and tyrosol, and many secoiridoids related to oleuropein like (7″*S*)-7″-hydroxyoleuropein, oleuropein 3′-*O*-*β*-d-glucoside, oleuropein-3″-methyl ether, jaspolyoside, jaspolyanoside, isojaspolyoside A, and ligustroside 3′-*O*-*β*-d-glucoside [26,27]. The antioxidant activity of these compounds was expressed as the antioxidant concentration in µg/mL required to reduce the concentration of the radical DPPH^•^ by 50% after 15 min of incubation (efficient concentration EC_50_). It was seen that between all compounds cited, oleuropein 3′-*O*-*β*-d-glucoside, jaspolyosid, and (7″*S*)-7″-hydroxyoleuropein had an EC_50_ value in the range of 46–100 µg/mL, which is higher than that of BHT (111 µg/mL) and lower than that of rosmarinic acid (6 µg/mL) [27]. Furthermore, hydroxytyrosol proved to be four times more potent than oleuropein and cycloolivil as a DPPH radical scavenger and higher activity than rosmarinic acid and, consequentially, than BHT [26]. These data agree with that of another study in which it was found that the efficient EC_50_ calculated for oleuropein and cycloolivil is higher than that of rosmarinic acid, hydroxytyrosol, vitamins E, and C but smaller than BHT [55]. This can be considered a good result since oleuropein is the most abundant compound present in solvent extracts of olive wood [54], as demonstrated in a study based on the evaluation of the antioxidant activity of 10 Spanish wood samples [31]. Overall, these compounds’ antioxidant activity may be related to the presence of the 1,2-dihydroxybenzene moiety in their structure. It is well-known, indeed, that the phenolic antioxidant activity effectiveness is linked to the phenoxy radical obtained by delocalization, which is why *ortho*-diphenolic compounds have enhanced activity, as the formation of *ortho*-quinone is favored [4].

The potential antioxidant activity of a compound is also indicated by its reducing power so that the transformation of Fe^3+^ to Fe^2+^, with ferric reducing antioxidant power (FRAP) test, and Cu^2+^ to Cu^+^, with cupric ion reducing antioxidant capacity (CUPRAC) test, in the presence of wood extract was investigated. It was seen that the wood extract exercised an intense reducing activity on either CUPRAC or FRAP [30], and this is in agreement with the results obtained when *O. europaea* pruning extracts were tested [43]. Tree pruning biomass (OTP) is a by-product generated by olive oil production, which is composed of thin branches (50% by weight), leaves (25% by weight), and wood or thick branches (25% by weight) [56]. OTP plays an important role within the biorefinery context as a raw material for producing fermentable sugars, oligosaccharides, antioxidant compounds, etc., [57,58]. In particular, the antioxidant properties of OTP are related to the presence of a high amount of flavonoids and polyphenols [44,59]. After the study of the optimal extractive conditions, it was seen that the greatest amount of TPC (total phenolic content) and TFC (total flavonoid content) was obtained by using ethanol in a percentage ranging between 50% and 56% [44,59]. Instead, about the extractive method used, the ultrasound-assisted extraction (UAE) seems to be the one that gives the best results in terms of active compounds extracted [59,60]. The flavonoids found in OTP includerutin, derivatives from chrysoeriol (glucoside) and apigenin (rutinoside), luteolin and its derivatives (mono and di-glucoside), the iridoid derivative loganic acid glucoside, and lignan (-)-olivil 4-O-β-d-glucopyranoside. The presence of the secoiridoidhydroxyoleuropeinwas also found, which, as previously said, is one of the compounds responsible for the antioxidant activity of wood extracts [44]. Furthermore, after OTP’s antioxidant properties evaluation by using three complementary methods such as DPPH, ABTS, and FRAP, the application of Pearson’s correlation confirmed a positive correlation between the antioxidant activity and either TPC or TFC [44]. 

Researchers have also studied the change in phenolic composition and, consequently, in *O. europaea* roots’ antioxidant properties under mycorrhizal colonization, water deficit condition, variation in soil salinity, and fluoride stress. The arbuscular mycorrhizal (AM) fungus is well-known to improve plants’ nutritional status. It was seen that the colonization of olive tree roots by AM fungi increased total flavonoid and phenolic content. The mechanism behind this evidence has not yet been elucidated. Still, it seems that the build-up of phenolic compounds in roots reflects a defense response by the plant against fungal invasion [22]. On the other hand, it was seen that root inoculation with mycorrhizal also result in changes in DPPH radical percentage inhibition since the highest percent of inhibition was evidenced in roots treated with AM [22]. It is in line with the increase of phenolic content; indeed, researchers have demonstrated that the decrease in the antioxidant activity reflects the reduction of phenolic content [61]. The water stress condition was even tested on olive roots. It was demonstrated that this condition determined an increase in oleuropein and a variation in other flavonoids glucosides such as luteolin-7 glucoside, luteolin-7-rutinoside, and apigenin-7-glucoside. It was hypothesized that in root exists a controlled system aiming to avoid serious oxidative stress induced by water deficit [62]. These results were in line with that obtained by inducing changes in salinity. Indeed, salinity was shown to stimulate phenol and oleuropein production, while no correlation was found in hydroxytyrosol concentration [63]. High saline water was also related to the downregulation of polyphenol oxidase (PPO) as a mechanism that olive trees developed to improve the phenols’ antioxidant action. Furthermore, alongside the downregulation of PPO, there was also an increase in antioxidant enzyme activity such as superoxide dismutase (SOD), ascorbate peroxidase (APX), and catalase (CAT) [64]. Hence, phenolic compounds’ build-up may represent an additional defense mechanism that olive trees adopt against oxidative stress. Contrarily, reducing antioxidant enzyme activity and mineral content and increasing oxidative stress markers like hydrogen peroxide were observed in an increased fluoride concentration known as the major contaminant present in the ecosphere [65]. Another study tested the freezing tolerance of olive trees by determining the bark DPPH scavenging activity. It was seen that the high level of DPPH scavenging capacity was related to an increase in freezing tolerance as a mechanism of defense against the overproduction of ROS [66]. Bark antioxidant activity can be associated with the high amount of phenolic compounds [67] since, like wood, it was rich in oleoperin and hydroxytirosol. Furthermore, from bark ethanolic crude extract were also isolated two compounds such as benzene-ethanol and 4-hydroxy-alcohol, with higher antioxidant activity (IC_50_; DPPH: 32.99 ± 0.20 µg/mL) than ascorbic acid (IC_50_; DPPH: 39.48 ± 0.02 µg/mL) [68].

In light of these results, it is possible to say that agricultural by-products from *O. europaea* contain different phytochemicals with a high antioxidant activity, which may offer a potential application in foods, pharmaceuticals, and cosmetics industries.

### 2.5. Agro-Industrial By-Products from O. europaea vs. Microbial Infections

Nowadays, drug resistance in microorganisms is endemic and constitutes a critical problem in healthcare delivery worldwide. Using the same drug for a long time has allowed microorganisms to acquire and transmit to each other different forms of resistance; hence, the emergence of pathogens with multi-drugs resistance now demands for the discovery of new active molecules. Several researchers have demonstrated that medicinal plants can cure several infections owing to their chemical composition since many secondary metabolites are generally produced in response to bacterial and fungal invasions. *O. europaea* has been used for years by different communities around the world to treat diarrhea, urinary and respiratory tract infection, intestinal and stomach diseases [69]. The anti-bacterial, anti-viral, and anti-fungal activity has been extensively studied for olive tree leaves, while only a small number of studies have been done on the antimicrobial activity of olive waste products. Thus, in this review, the knowledge acquired to date on the antimicrobial activity of *O. europaea*’s roots, bark, wood, and pruning (stems or twigs) has been exposed. Makirita W.E. et al. have investigated the effect of olive root extract on different Gram-positive and Gram-negative bacteria. It was demonstrated that olive roots methanolic extract had a MIC (minimum inhibition concentration) value of 0.7812 mg/mL against *Pseudomonas aeruginosa,* and the same MIC was evidenced for stem bark methanolic extract against *Proteus mirabilis*. Furthermore, roots methanolic extract possessed a MIC value of 1.5625 mg/mL against *Klebsiella oxytoca*, *Klebsiella pneumoniae*, and *Salmonella typhi* [70]. The bark extract’s inhibitory effect on bacteria was confirmed in another study, even if the same activity was not demonstrated on fungi. In a particular way, it was seen that ethanolic bark extract showed high inhibitory activity against Gram-positive bacteria like *Bacillus subtilis* and *Staphylococcus aureus* (12.33 ± 0.66 mm and 12.00 ± 0.58 mm, respectively). In contrast, only moderate activity was evidenced against Gram-negative bacteria such as *Escherichia Coli* (10.67 ± 0.33 mm) [71]. The findings show that Gram-negative bacteria are usually less sensitive to plant extracts than Gram-positive because of the presence of an outer membrane that acts as a barrier to biomolecules’ entry [72]. 

The anti-bacterial and anti-fungal activity was even investigated for olive wood extracts, which demonstrated different degrees of antimicrobial properties depending on the extract amount, extract samples, and bacterial strain. It was shown that olive heartwood extract did not affect *Salmonella Kentucky*, *Enterobacter aerogenes*, and *Salmonella typhimurium*. However, olive sapwood extract has demonstrated a high effect on *Listeria monocytogenes* with an inhibition zone of 26, 25, and 16 mm using 1.6, 1, and 0.6 mg of the extract, respectively. Furthermore, either heartwood or sapwood extracts have demonstrated anti-fungal activities against Candida albicans using 1.6 mg of the extract [30]. This anti-fungal activity was even demonstrated for the same extracts on *Pleurotusostreatus* fungi whose growth was inhibited by approximately 80% [29]. Another by-product of *O. europaea* wood was also tested, namely the oil tar obtained from cutted wood’s distillation. It was seen that the efficacy of wood tar oil was higher than that of streptomycin. Indeed, its inhibition rate of the growth of bacteria reached around 16.33 to 46.00 mm. Specifically, the greatest susceptible strain was *Pseudomonas aeruginosa*, followed by *Micrococcus luteus* (inhibition zone diameters were 46.00 mm and 45.33 mm, respectively), while the greatest resistant bacteria was *Staphylococcus aureus* which possessed a growth area inhibition of 16.33 mm [25]. Another study also demonstrated that wood tar oil is active against *Klebisiella pneumoniae* with an inhibition growth diameter of 34 mm [73]. The MIC values assed are 0.032 mg/mL for *Klebisiella pneumoniae*, 0.05 mg/mL for Staphylococcus aureus, 0.1 mg/mL for *Pseudomonas aeruginosa*, *Enterococcus faecalis*, *Escherichia coli*, *Listeria monocytogenes*. Furthermore, in concern for fungi, it was shown that wood tar oil has a high anti-fungal activity with a growth inhibition rate that ranged between 0.006 and 0.1 mg/mL.The greatest inhibition rate was seen for *Fusarium oxysporumf spalbedinis* (0.006 mg/mL) [73]. These anti-fungal and anti-viral properties can be related to several phenolic compounds found in the wood extract, including hydroxityrosol, oleuropein, tyrosol, protocatechuic acid, vanillin, *p*-cumaric acid, and benzoic acid. In fact, many studies have demonstrated their anti-bacterial and anti-fungal activities [30].

Finally, olive stems or twigs extracts were investigated for their anti-viral activity against coxsackievirus B-3 (CVB-3) and herpes virus type 2 (HSV-2). It was demonstrated that the hexane fraction of twigs extract exhibited an anti-viral activity only against CVB-3 (concentration cytotoxicity 50% value around 100 μg/mL), while no activity was observed for HSV-2. The compound 2,4-di-*tert*-butylphenol, isolated by TLC and identified by GC-MS, is responsible for this activity. This is a molecule produced by microorganisms, such as *Streptomyces* sp. and *Lactococcus* sp., present in several plants and has been demonstrated to have also activities against white spot syndrome virus (WSST) and yellow head virus (YHV) [45]. Considering the above, it is possible to say that *O. europaea* by-product can be considered a source of alternative compounds for treating several infectious diseases. Nevertheless, further investigations are required to identify the major bioactive molecules responsible for these olive tree extracts’ antimicrobial and antioxidant action.

### 2.6. Agro-Industrial By-Products from O. europaea and Other Biological Activities

As previously said, the health benefits of olive trees agro-industrial by-products have been poorly studied. Only a small number of papers are present in the literature. In particular, the cardiovascular and anticancer activity of olive root, wood, bark, and stem has been investigated. Recently platelet antiaggregant properties of oleuropein and (+)-cycloolivil isolated from the olive wood extract were evaluated. In particular, it was found that these two molecules were able to attenuate platelet aggregation stimulated by thrombin in platelets derived from either healthy or type 2 diabetic patients. Moreover, since for platelet aggregation the mobilization of Ca^2+^ is essential, the effect of oleuropein and (+)-cycloolivil on agonist-evoked Ca^2+^ signaling was investigated. It was seen that both molecules were able to attenuate the mobilization of Ca^2+^ stimulated by thrombin (a physiological agonist of Ca^2+^ mobilitation) in platelets of either healthy or type 2 diabetes patients, although, in this last group, the effect was more significant [55]. It is known that, in diabetic patients, ROS plays an important role in inducing the dysfunction of Ca^2+^ homeostasis [74], so that the effect of oleuropein and (+)-cycloolivil may be related to their capacity in reducing oxidative stress. Another factor essential for platelet aggregation [75] and so for the mobilization of Ca^2+^ in these cells [76] is the protein tyrosine phosphorylation. It has been reported that oxidative stress plays a role in modulating many protein kinase/phosphatases [45]. It was demonstrated that both oleuropein and (+)-cycloolivil were able to attenuate protein tyrosinase phosphorylation. Further, this effect is greater in diabetic patients than in healthy volunteers so that, after phenolic compounds assumption, the platelet proteins phosphotyrosine content was similar in both diabetic control groups [55]. In addition to these findings, there are those derived by an in vivo study where oleuropein isolated by olive roots extracts was investigated against the cardiac remodeling process after infarction induced by isoproterenol in rats. It was demonstrated that pre-cotreatment with oleuropein decreased the heart weight index (HWI) and avoided cardiac tissue hypertrophy in isoproterenol-induced myocardial infarction in rats [77]. It is known that an excessive heart weight may be attributed to the increase in water content, necrosis of fibers of cardiac muscle, and excessive adenomatous intramuscular space leading to the invasion of the damaged tissue by inflammatory cells [78]. Treatment with oleuropein was shown to reduce edematous in the myocardium, as observed in histological samples taken from mice [77]. Further, oleuropein evidenced a positive effect on cardiac rhythm with a dromotropic effect, reduced isoproterenol-induced serum diagnostic serum marker enzyme (tropin-T, LDH, ALT, and CK-MB) elevation, and also inhibit the angiotensin-converting enzyme (ACE) activity. In fact, it was demonstrated that the cardiac renin-angiotensin system (RAS), formed by angiotensin II, ACE, and angiotensin II receptors, was activated by cardiac remodeling processes occurring during acute myocardial infarction [79,80]. This effect led to increased serum ACE activity with a consequent remarkable decrease in systolic, diastolic, and arterial pressure. Oleuropein exerted ACE activity inhibition with the resulting restoration of the hemodynamic function in isoproterenol-induced infarction in rats. Another effect of isoproterenol-induced infarction in rats was a significant rise in serum lipase activity, leading to the increased serum of total cholesterol, triglycerides, and LDL levels with a decrease in HDL. These alterations in lipid profile may be due to the cardiac cyclic adenosine monophosphate, which was found to be responsible for the enhancement in lipid biosynthesis during the hypoxic condition revealed in isoproterenol-induced infarction in rats. Oleuropein is considered a good inhibitor of lipase activity, as demonstrated by the improvements in serum lipids profile [77]. Thus, in the light of the above, olive wood and roots extract may protect against cardiovascular complications associated with several diseases like type 2 diabetes mellitus and hypertriglyceridemia and deserves to be studied more extensively. 

Recently *O. europaea* steam extract was also tested for its anticancer activity on human melanoma cells. Multiple myeloma is a hematological disorder representing 1% of all malignancies characterized by cells that usually acquired the capacity to inactivate cell death pathways, leading to death avoidance. This feature often ends in developing resistances to chemotherapies, so researchers have moved to the investigation of new anticancer molecules able to induce apoptosis and hence tumor cell death. The olive steam extract (OSE) had demonstrated strong anti-multiple melanoma properties since it induced anti-proliferative effects in a dose-dependent manner on U266 myeloma cells. In particular, it was seen that the exposure for 24 h to OSE led to cell cycle disruption represented by a significant increase in sub-G1 phase, a decrease in the G1-phase, and an accumulation of cells in S phase [81]. This is in agreement with other studies that demonstrated the ability of oleuropein in inhibiting breast cancer cell proliferation by stopping the cell cycle at the S phase [82]. Further, it was demonstrated that molecules as hydroxytyrosol, oleuropein, and verbascoside, abundant in olive bark, were able to act as a poison for the Topoisomerase II, avoiding cell entry in mitosis and freezing the cell cycle in the S or G2 phase [83]. OSE also induced apoptosis in U266 myeloma cells as demonstrated by the cell morphological changes, including chromatin condensation, nucleus fragmentation, and formation of bodies enclosed in a membrane and containing organelles confirming the ongoing apoptosis. At the basis of this process may be the activation of the intrinsic apoptotic pathway since OSE had shown the ability to induce caspase 3/7 within 24 h of treatment [81]. The activation of apoptosis evaluated via morphological cell change was even demonstrated after the treatment of immortalized keratinocytes with oleanolic acid, a triterpenoid found in roots of *O. europaea* [84]. All these results are promising and may form the basis for further investigation of extracts’ anticancer activity from olive by-products.

### 2.7. Industrial Uses

**Figure 6:**
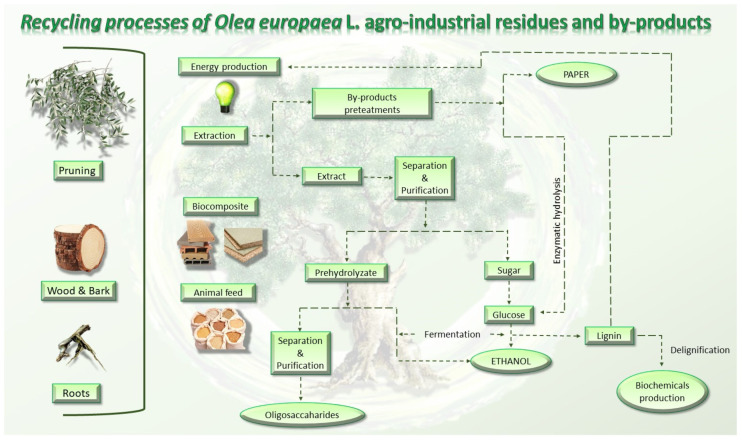
Graphical representation of *O. europaea* agro-industrial by-product recycling processes.

The present section summarizes information given by the scientific literature on *O. europaea* agro-industrial by-product recycling processes (Figure 6).

#### 2.7.1. Animal Feed

Since considerable amounts of olive by-products like pruning residues are generated from the olive trees management, many research focus on their valorization. Among different wood species such as oak and vine branches, olive trees are considered a source of nutriment for sheep and cattle during grazing in Spain. However, fibers and tannins within these products limit their nutritional value since they may lead to a decrease in digestibility. Using the summative equation of digestibility prediction based on plant lignin and fiber contents, researchers estimated the in vitro nutritional value of these by-products used as animal feeds. This was evaluated by using the sulfuric lignin content of fiber rather than permanganate lignin. The condensed tannins inclusion into the predictive equation, considered as an element that may cause a digestibility decline, improved the prediction of the in vitro value from chemical composition [85]. Further, determination of the in vitro digestible organic matter (IVDOM), metabolizable energy (ME), net energy lactation (NEL), and presence of nutritional and anti-nutritional components was carried out to evaluate the nutritional values of branches from *O. europaea* trees cut at 25, 50, 75, and 100 cm distance from the tip. The addition of polyethylene glycol (PEG, 6000) to the plant samples incubated with rumen fluid at a ratio of 2:1 (PEG: substrate) showed an increase in the values of IVDOM, ME, and NEL by 40 g/kg DM, 0.59 MJ/kg DM, and 0.42 MJ/kg DM, respectively. It is possible to explain these results based on the knowledge that PEG forms complexes with tannins [86] and can reduce tannin–protein complex formation or disrupt the link between these complexes [87].

Another study has demonstrated that olive pruning branches in diameter < 3 mm could be used as feeds for small ruminants belonging to the Mediterranean zones [88]. This would be possible owing to the high degradability, partitioning factor, as well as microbial nitrogen. Further, following the previous study, the values of rumen microbial nitrogen, fermentation characteristics, and degradability of dry matter increased by adding polyethylene glycol to the olive pruning branches [89]. Hence, based on these results, it is possible to say that pruning residues may provide the fulfilment of the animal nutritional needs.

#### 2.7.2. Sustainable Composites for the Engineering Sector as an Example of Waste Management

Olive residues are largely used in many biomass power plants present in the Southern part of Spain (Andalusia). Power stations are focused on energy production, and it is possible to distinguish two types of residues: biomass fly ash (BFA) and biomass bottom ash (BBA). BFAs are generated from particles that are washed away by the gas stream to the outside of the combustion chamber and used in fertilizers to enhance the natural fertility of the soil. On the other hand, BBAs are formed by particles not combusted [90], which are transported to landfills because the properties of these residues related to the mechanical behavior in civil applications are not well-known. BFA was the focus of a study conducted in Spain to analyze the effects of olive residue biomass fly ash as filler in self-compacting concrete instead of cement content. It was found that the compressive strength in concretes manufactured replacing the Ordinary Portland cement with BFA resulted in being slightly higher than those made with conventional filler. From this result, it can be stated that fly ash from the combustion of agricultural olive residue pellets is suitable as a filler in high-quality self-compacting concrete manufacturing [90]. Beltrán et al. (2014) took into account the use of BBA from the combustion of olive tree pruning as an alternative to cement in non-structural recycled concrete. The addition of the BBA replacement led to a significant decrease in mechanical and durability properties in the manufacture of recycled concretes [91].

Besides the involvement of the olive pruning residues ash generated by biomass power plants for recycled concrete manufacture, there are studies concerning alternative methods for recycling agricultural residuals in recent years. Biomass-based materials for building applications have been studied to obtain environmental-friendly products. This is related to the increasing pressures on the use of more sustainable materials capable of improving indoor environment quality and reducing energy consumptions. Liuzzi et al. (2020) [92] affirm that olive pruning waste (leaves and small branches) combined with barley straw fibers and bonded with a low-toxicity agent (sodium silicate solution instead of polystyrene that uses benzene derived from petroleum), could be suitable for the indoor covering panels production with hygrothermal and acoustic properties. In another study, the same authors stated that adding a different percentage of leaves and small branches derived from olive trees pruning in clayey mixtures had a strong effect on thermal conductivity properties. The increase in olive fibers percentage leads to an enhancement of the thermal insulation capacity [93]. Research conducted in Spain has evaluated new formulations to improve thermal insulation and mechanical properties of ceramic bricks made of clay mixed with olive waste (pruning residues, leaves, and wood) at different percentage ratios (7.5%, 15%, and 25%) and compared with clay bricks without waste products of olive cultivation. Generally, ceramic brick is used as a building material made of red clay by molding and firing. The best finding of this research was obtained from the samples with a percentage ratio of olive waste of 7.5%. The conclusion is that these organic pore-forming wastes may be considered a valid alternative to produce lightweight ceramic bricks with acceptable mechanical properties together with a high thermal insulating capacity [94]. In another study, olive wood in the form of dust was mixed with a polyactic acid (PLA) filament to make three-layered sandwich beams using 3D-print technology for lightweight structures in the building sector [95].

A type of material whose lightness and enhanced mechanical performance have resulted in an increasing number of applications in building, construction, and furniture, is wood-polymer composite (WPC). It consists of two main constituents, both polymer-based: the plastic, which is the matrix of the composite (polyethylene, polypropylene, polystyrene, polyvinyl chloride, polyethylene-terephthalate) and wood fiber (reinforcement) with some bonding agent [96]. WPC based on polypropylene integrated with olive wood fibers as a filler, was studied to increase the inflexibility of the resulting composite material. In particular, the effect of the wood flour content and its chemical fiber treatment (amino-silane) on the mechanical properties of WPC was analyzed through ultrasonic methods and mechanical tensile tests. The ultrasonic evaluation was applied as a method to assess the elastic properties of the WPC. The rigidity of composite materials increased with increasing fiber content and the addition of the amino-silane coupling agent [97,98]. Moreover, among the various polymers dominating the area of polymers for WPC, polyvinyl chloride filled with wood flour derived from the olive tree has been studied in terms of hydrothermal ageing effects on thermal behavior and then compared with unfilled polyvinyl chloride. The authors concluded that the material thermal stability decreased with the addition of wood flour. Further, results from hydrothermal ageing of both sample types (unfilled and filled ones) showed that all of these absorbed water. This water absorption causes a decrease in thermal stability. Generally, natural fibers have the disadvantage of being hydrophilic, and, at the same time, the thermal degradation at high temperatures makes their processing problematic. Natural fibers degrade above 200 °C but, in this study, it was found that the filled samples can be easily processed industrially at temperatures between 180 and 200 °C without damage [99]. Fiber extracted from natural resources can be used as reinforcement in a polymer composite, as stated by Alshammari et al. (2019) [35]. In fact, the object of their research refueled fibers extracted from leaves, small and large branches coming from olive trees, and it was demonstrated that small and large olive branches are thermally stable compared to olive leaf fibers. Hence, it is possible to say that these natural fibers can be part of a new polymer composite development for different lightweight applications.

All the studies above mentioned demonstrated the vastness of the possible ways to recycle wastes coming from olive trees pruning showing the progress toward the eco-efficiency of the construction industry.

#### 2.7.3. Olive Tree Pruning vs. Biorefinery

The management of biodegradable organic waste is also turning into a different approach that involves creating a new industrial supply chain, of so-called biorefineries [100]. Biorefinery consists of generating an industry based on value-added products deriving from biological materials obtained through valorization/conversion processes of the different biomass components (polysaccharides such as cellulose, hemicelluloses, lignin). Processes including autohydrolysis [41], steam explosion [34], alkaline peroxide delignification [34], organosolv pretreatment [101], dilute acid hydrolysis [102], hydrothermal pretreatment, pulping process with ethanol combined with saccharification, and fermentation process [37,38]. Totally chlorine free (TCF) bleaching [103], has been tested for fractionating olive tree pruning residues. It has been reported that biomass from olive tree pruning is suitable for the production of oligosaccharides, and is considered important because of their wide-ranging application in feed, food, pharmaceutical, cosmetics, and agrochemistry industries [104]. Fractionation and purification of oligosaccharides by olive tree pruning autohydrolysis in the range 170–230 °C has been assessed. Under conditions of 180 °C of temperature, it was possible to obtain the highest oligosaccharide yields [41].

Olive prunings are considered a potential lignocellulosic feedstock to produce energy and other value-added products as an alternative to starch-containing feedstocks. From an economic standpoint, it is crucial to recover sugars from hemicellulose. The use of dilute acid can lead to rapid hydrolysis conditions, providing d-glucose- and d-xylose-rich hydrolysates that do not require further processing. The effect of residence time, temperature, and sulfuric acid concentration on the formation of d-glucose and d-xylose was estimated using the response surface method. Batch hydrolysis was performed at very low temperatures (70–90 °C) and concentrations of H_2_SO_4_ from 0 to 1 N, sampling at different times from 0 to 300 min. According to the statistical analysis, all three parameters had significant interaction effects on sugar production. Results illustrated that the highest concentrations of d-glucose and d-xylose were found at the highest temperature, acid concentration, and residence time analyzed. Under these conditions, the maximum expected yields expressed as g of sugar per 100 g of fed dry matter were 0.13 in d-glucose (about 40% of the maximum achievable) and 0.10 in d-xylose (about 60% of the potential yield). Under these conditions, about 40% of the maximum achievable d-glucose and about 60% of the potential d-xylose were predicted to be obtained. On the opposite hand, no sugar degradation was evidenced. Therefore, the sugar-rich hydrolysate obtained from this widely available renewable agricultural residue by dilute acid hydrolysis could be fermented to obtain ethanol or other value-added products [105].

Bioethanol coming from the fermentation of agricultural products rich in sugars is used as liquid biofuel and can be produced from olive wood. Specifically, steam pretreatment using a temperature of 190 °C followed by alkaline peroxide delignification and enzymatic hydrolysis gave the highest sugar yield usable for bioconversion into ethanol [34]. For the first time, Dìaz et al. (2011) [101] describe the organosolv pretreatment on olive tree pruning biomass, taking advantage of its richness in sugar content. To access its sugar content, the mentioned pretreatment was required to improve enzymes accessibility of the residue cellulose fraction. Fractionation of olive tree pruning biomass using organosolv pretreatment enabled enzymatic sugar production. Olive tree pruning was separated into two fractions: the main fraction including stems and trunks with a diameter >1 cm and the residual one including leaves and stems with a diameter < 1 cm. Both fractions have undergone biorefinery procedures. It was possible to obtain pulp for paper or bioethanol production from the main fraction and the residual fraction as fuel [38]. Lignin is the branched polymer present in plant cell walls and, together with cellulose, constitutes the cell membrane. It was extracted from olive tree pruning through organosolv pretreatment to evaluate phenolic monomer compounds. Syringol and guaiacol dealkylation, demethoxylation, and demethylation were crucial to recover the highest phenolic compounds (phenol, o-cresol, m-cresol, p-cresol, catechol, and 4-methylcatechol) yield to comply with the enormous industrial demand for aromatics [40,106].

#### 2.7.4. Olive Wood Residues in the Renewable Fuel Sector

Pellets are considered sustainable energy fuels. The measurement of the quality of pellets for domestic use by olive tree pruning follows the CEN/TS 14961:2005 parameters published by the European Standard Committee CEN/TC 335. The data showed that the energy density parameters of olive pruning residues (OP), which influence the combustion process, and the bulk density values, were similar to those found in the literature on other woody species. The moisture content of OP is 5.37%, in line with the values allowed by the regulations for domestic use pellets. Regarding the crushing resistance values equal to 22% of the average value, even if not present in the standard, provides a quick measure of the pellet quality. About the tensile strength, the samples of olive pellets showed an average value of 176 numbers per 100 g. This parameter is also not included in the standard but is linked to the strength and density tests. Another important parameter to evaluate is the pellet size which influences the combustion process of the same. OP pellets size is around 6 mm and there is a correlation between the length of the pellets and the density of their particles. Pellets from olive pruning residues (OP) are more densified and therefore broke less frequently. Again, the olive pellets were found to be in line with the normative. The chemical composition of the pellets showed a high content of nitrogen (<0.3%) compared to what is allowed by law. Concerning the limits of additives use required by the regulations, as no additives were added to the pellets, this parameter is in line with the rules. The ash content showed values higher than those required by law (≤0.7%), probably due to the presence of sand and soil. The last important quality parameter evaluated was the calorific value. This is in line with the parameters present in the literature and in line with the parameters present in the regulations for household pellets [107].

The factors that may influence olive wood pellets and pruning quality were analyzed indepth through the methodology described in the European Standard EN 14961-2. Table 2 shows the rules to follow when producing pellets for non-industrial use, while Table 3 gives an overview of the characteristics of pre-pelletizing materials.

It was found that the hardness of olive wood facilitates the reduction of pellet particle size to approximately 2.5 mm. In terms of chemical properties, analyses of olive wood pellets showed that the raw materials exceeded the values for ash, nitrogen, and sulfur that the guidelines established for non-industrial pellets. To solve these limitations, it would be necessary to mix different feedstocks to reduce the percentages of these chemical components in the final biomass samples.

According to the guidelines of EN14961-2, after the pelletization process of olive wood materials (pruning and wood), it can be seen that the pellets have the characteristics reported in Table 4.

After the pelletizing tests, it was noted that the OW samples met the requirements set out in EN 14961-2 for non-industrial pellets; in the case of OP pellets, they could not be selected as optimal OP samples because they had a bulk density of less than 600 kg/m^3^. From the selected samples, it can be concluded that a low moisture content before pelletizing (9%) is more suitable for producing pellets from different types of olive trees’ residual biomass, while the compression length and pelletizing temperature must be adapted to the biomass to be pelletized. Short compression lengths are suitable for producing high-quality pellets from olive trees’ residual biomass. In the case of OP, temperatures above 60 °C are needed to improve pellet quality, while temperatures in the range of 40–60 °C are sufficient to produce pellets from OW [108]. In detail, the analysis of olive pellets quality was studied through the analysis of process parameters such as temperature and pressure, through the characteristics of the biomass of moisture and size, and the mechanical properties such as density and durability. The technical specification UNI/TS 11263:2007 defines clear principles to characterize the quality of pellets for energy purposes easily. As in the previous studies, the diameter of the pellets was around 6 mm, precisely ranging from 6.03 to 6.31 mm, the length was between 16.63 and 27.83 mm, the pellet mass varied between 0.60 and 0.70 g. The analysis showed that the density and hardness of the pellets are determined by the following factors: high temperature, low moisture content, and small particle size. Modulus of elasticity and density are little affected by applied pressure. It was also seen that there is a strong correlation (>80%) between density and compressive strength, density and modulus of elasticity, compressive strength and modulus of elasticity. From a practical point of view, the parameters for producing quality pellets are the temperature around 150 °C, a low applied pressure (70 MPa), a high moisture content h (20% w.b.), and biomass ground with a 4 mm hammer mill. If applied to industrial plants, these parameters can reduce the energy needs of the pelletizing procedure [109].

From these first research on the quality of pellets for domestic use and non-domestic use, several critical points on the use of these raw materials have emerged, but further research is necessary to obtain good quality pellets using these raw materials.

It is also important to analyze wood chip combustion and emission behavior of different agricultural biomasses, including olive tree woody residues. Measurements were conducted in the laboratory and the emission of carbon monoxide (CO), carbon dioxide (CO_2_), oxygen (O_2_), nitrogen oxides (NOx), sulfur dioxide (SO_2_), total organic compounds (TOC), and particulate matter (PM) was measured during combustion in a 30-kW boiler equipped with a multi-cyclone bag filter for emission abatement. In this study, it emerged that among the biomasses analyzed, olive residues are considered the most advantageous biomass from every point of view, both in terms of emissions and energy efficiency. Another advantage is the widespread use of olive tree pruning and its high availability, leading to a real sustainable production chain that respects emission limits. The high availability of pruning and good energy behavior make the olive tree the best solution in terms of fuel use in biomass plants, together with devices that can control and reduce the emission of pollutants [110].

#### 2.7.5. Olive Wood Residues as Active Carbons

Activated carbon may be a material containing primarily amorphous carbon and having an extremely porous structure and high specific space. Due to the high specific space, activated carbon is able to retain several molecules of alternative substances within it, having the ability to accommodate these molecules on its giant internal area. Therefore, activated carbon may be a material with high adsorbent capability. Activated carbon is employed in the filtration, purification, deodorization, and decolorization of fluids. There are two strategies for producing the chemical activation of carbon supported by some chemical compounds’ dehydrating action, like oxygen acid or metallic element chloride. The temperature at which this occurs is between 400 and 1000 °C. Gas activation suggests that of a vaporized mixture containing chemical elements or dioxide. At a temperature of 800–1000 °C, a number of the beginning materials decompose, producing various extraordinarily small pores and cracks.

Analyses were carried out using olive wood residues as activated carbons, activated by KOH and H_3_PO_4_. Results showed a very high surface area with a well-developed micro-and mesoporosity with several surface functional groups from the tests performed. Electrochemical tests in 1 M H_2_SO_4_ indicate that the sample prepared from olive KOH had discrete electrochemical characteristics with a capacitance of 257 F g^−1^ and energy density of 4–2 Wh kg^−1^ at a power density of 47 and 1400 W kg^−1^. While the samples activated with H_3_PO_4_ showed high phosphorus content, which improved their electrochemical characteristics but gave them lower stability than those activated with KOH at various power densities. Olive samples showed very interesting stability with high capacitance, energy, and density, which is due to the volume of micro- and mesopores and their surface functional groups. The stability of the electrodes against charge and discharge cycles shows that the carbons from olive wood residues activated with KOH and H_3_PO_4_ have excellent stability [111].

Ouldriss [12] also analyzed the formation of activated carbons from woody residues activated not only with H_3_PO_4_ but also with air. In agreement with the previous study, olive wood residues showed a discrete porous development. The obtained results clearly showed that the samples prepared at 350 and 400 °C possessed good porous development. In contrast, once the carbonization temperature increased on top of 450 °C, a well-developed mesoporosity was observed. This is attributable to the tendency of the complex mixture of phosphorus species present in the lignocellulosic substrate impregnated with phosphoric acid to evaporate. This tendency leads to an expansion of the samples’ internal structure, thus producing a well-developed mesoporosity in these samples. Mercury intrusion curves indicate that the samples show a significantly developed mesopore volume as well as a wide variety of mesopores ranging from 40 up to 1100 Å in diameter. Thus, if the proper conditions are used, it is possible to prepare activated carbons that exhibit specific micro- or mesoporous structure and surface area properties. Further, it was seen that the operating conditions used for sample preparation greatly influenced the development of surface area and structure of activated carbons prepared from olive wood by chemical activation. If these conditions were carefully controlled, it was possible to obtain carbonaceous materials with tailored porosity and surface areas. The samples showed textural properties that could be extremely useful for the removal of organic and inorganic pollutants in an aqueous solution. It can be concluded that the chemical activation method leads to samples that exhibit a well-developed porous structure.

Concerning the results obtained on the preparation of activated carbons by physical activation of olive wood with air, it was seen that the prepared samples show significant values of specific surface area and volumes of micro-, meso-, and macropores. A significant difference emerged between the S_BET_ (specific surface area) and pore volumes depending on the treatment time for which the activation process is performed. For samples activated for 0.5 h or 1 h, the surface area and texture development were remarkable, although less than those obtained when the treatment time was increased to 2–3 h. This fact could be attributed to the progressive destruction of the more labile parts of the lignocellulosic substrate, which was associated with a more notable presence of micro- and mesoporosity in these samples. The samples exhibit textural properties that could be extremely useful for the removal of uncharged organic pollutants in an aqueous solution. Moreover, in the case of the preparation of air-activated carbons, it can be concluded that the physical activation method resulted in samples showing a well-developed porous structure [112].

#### 2.7.6. Olive Wood Residues as Paper

In recent years the production of paper from woody residues in a circular economy perspective has gained increasing interest. There are several studies conducted on the production of pulp and paper sheets obtained from olive wood residues. To pulp woody residues, there are three different processes. In particular, these processes are mechanical, chemical, and, finally, semi-chemical. The chemical pulping process is very popular today and is mainly done using the “Kraft process,” in which caustic soda and sodium sulfate are used to convert wood chips into dark brown pulp.

The efficiencies of soda ash, sulfite, and kraft pulping processes applied to Iranian olive wood were compared. The influence of pulp beating on drainage (Shopper-Riegler index) and the properties of paper sheets obtained from the pulps (break length, elongation, burst index, and speed) were examined. Sulfite pulp showed the highest degree of whiteness (41%). The difference in brightness between Kraft and soda paste was not significant. The soda paste required more intense beating than either the kraft or sulfite pastes. Kraft pulp exhibited the highest holocellulose to yield ratio, while sulfite pulp soda pulp exhibited higher lignin to yield ratio. The holocellulose content per unit yield of pulps from olive wood subjected to the kraft and sulfite processes exceeded that of the soda pulp by 15.2% and 13.7%, respectively. The pulp-to-yield ratio of kraft and sulfite pulps is 15.9% and 8.7% higher than that of soda pulp, respectively. On the other hand, the lignin/yield ratio of soda pulp is 11.4% and 36.1% higher than that of kraft pulp and sulfite, respectively. All this is reflected in higher sheet strength from kraft and sulfite pulps and higher brightness in sulfite pulp. Soda pulp requires more intense beating than kraftand sulfite pulps, and PFI beater must be operated at 50% higher beating speed to obtain pulp with 80–85° SR. On the other hand, the lignin/yield ratio of soda pulp is 11.4% and 36.1% higher than that of kraftand sulfitepulp, respectively. All this is reflected in higher sheet strength from the kraft and sulfite pulps and higher brightness in the sulfite pulp. With similar beating, olive wood kraft and sulfite pulps provide paper sheets with higher breaking length, elongation, burst index, and tear index than soda pulp of the same material. Sheets of paper from kraft with breaking length 5851 m, elongation 4.61%, burst index 4.48 KN/g, and tear index 2.03 mN m^2^/g, showed the highest strength [33].

The effect of processing variables (temperature, pulping time, and soda concentration) on the properties of the pulp (holocellulose, a-cellulose, and lignin content, yield, and brightness) and the resulting papers (break length, bursting, breaking strength, and heat resistance) was studied. To obtain a pulp with acceptable yield, holocellulose and a-cellulose content, high brightness and low lignin content, cooking at a low pulping temperature about 155–160 °C was required, using a high concentration of soda ash (10%) for a short pulping time (15 min). However, obtaining paper sheets with acceptable strength properties requires quite different conditions. Consequently, it is preferable to use different pulping conditions that lead to the best pulping properties and improve the paper sheet characteristics with refining [113]. It was measured by sulfite pulping additional process variables of olive wood such as temperature, pulping time, sulfite concentration, anthraquinone concentration and liquid/solid ratio, properties of the pulps (yield, holocellulose, a-cellulose, and lignin content, and brightness) and the paper sheets (elasticity, burst index, and tear index) obtained. To achieve pulps from sulfite pulping with acceptably high yield, holocellulose content and a-cellulose content, and also high brightness, as well as low lignin content, it is necessary to operate at a temperature of 193 °C for 143 min, using a sulfite concentration of 19.85%, an anthraquinone concentration of 0.1%, and a liquid-to-solid ratio of 6.24. Using these parameters, paper sheets with high elongation, burst, and tear index can be obtained [114]. Effect of process variables such as temperature, pulping time, and ethanol concentration on the properties of the pulp produced (yield and content of holocellulose, a-cellulose, and lignin) and the pH of the resulting wastewater in the production of ethanolic pulp from olive tree trimmingswas also studied. Obtaining pulp with an acceptably high yield (37.6%), high holocellulose and a-cellulose contents (above 88.8% and 46.9%, respectively), and low lignin content (below 7.2%), involves operation at a pulping temperature of 200 °C, an ethanol concentration of 75%, and a pulping time of 60 min. Ensuring optimal pulp composition (e.g., high holocellulose and a-cellulose content, and low lignin content), involves the use of a high temperature and ethanol concentration, as well as a long pulping time. It was noted that decreasing the values of the process variables increases the yield and pH of wastewater but has no appreciable effect on holocellulose, a-cellulose, or lignin contents relative to their optimal values (95.9%, 50.7%, and 5.8%, respectively). These operating conditions are advantageous for the raw material characteristics, saving ethanol and time, and the wastewater had a pH of 4.29, which is not too different from the maximum pH served (5.11) [115]. The influence of independent variables in pulping olive wood cuttings using ethanolamine-soda-water mixtures (i.e., cooking temperature 165–195 °C) and time (30–90 min), ethanolamine concentration (5–15%) soda ash concentration (2–15%), ethanolonolamine concentration (5–15%), soda ash concentration (2.5–7.5%), and liquid-to-solid ratio (4–6), on yield, viscosity, and holocellulose, a-cellulose, and pulp lignin contents was analyzed. Obtaining a pulp with the highest possible holocellulose content, a-cellulose content, and viscosity, i.e., 84.0%, 68.2%, and 697.5 mL/g, respectively, and the lowest possible lignin content (11.4%) involves the use of high concentrations of ethanolamine and soda ash, and also high temperature (except if the holocellulose content is to be maximized, in which case it should be 187 °C). Moreover, a long cooking time is necessary (to maximize viscosity and minimize lignin content); and a medium, high, and low liquid-to-solid ratio for optimal a-cellulose, viscosity, and lignin content, respectively is required. The pulp was obtained using high concentrations of ethanolamine and soda ash, and high temperature, short cooking time, and low liquid-to-solid ratio. It shows values of holocellulose, a-cellulose, and lignin content and a viscosity that differ by less than 0.8, 3.2, 8.8, and 14.7%, respectively, from the optimum values and a pulp yield that is only 22.6% lower than the maximum value (52.2%). These operating conditions result in capital savings through the use of smaller structures, shorter cooking times, and reduced amounts of liquid. The use of a 15% ethanolamine concentration, a 7.5% soda ash concentration, and a liquid-to-solid ratio of 4 at 195 °C for 30 min will provide pulp with near-optimal holocellulose, a-cellulose, and lignin loss without unduly affecting yield and viscosity [116]. The influence of independent variables in the ethylene glycol/soda pulping of olive wood waste (165–195 °C, 30–90 min), with ethylene glycol concentration 5–15%, soda concentration 2.5–7.5%, and liquid/solid ratio 4–6, on the yield and strength properties (breaking length, burst index and tear index) of the paper sheets was analyzed. Using a temperature of 184 °C, ethylene glycol and soda ash of 15% and 7%, respectively, a liquid-to-solid ratio of 5:1 and a baking time of 30 min results in yield, rupture length, burst index, and tear index, which deviates by 14.3%, 17.1%, 17.0%, and 2.3%, respectively, from their optimal levels. These conditions result in substantial savings in energy consumption and capital investment tied up as they result in a lower temperature, lower liquid-to-solid ratio, and shorter time than the maximum values tested. Pulp production with ethylene glycol and soda ash blends produces pulp with higher yields, somewhat poorer strength properties for the resulting paper than those obtained with ethylene glycol or soda ash alone. Based on the high values obtained olive wood is not suitable for use with this pulping process if the resulting pulp is to be subsequently subjected to bleaching [117]. The independent variables in ethylene glycol/soda pulping of olive wood cuttings [i.e., cooking temperature (165–195 °C and time (30–90 min)], with ethylene glycol concentration (5–15%), soda concentration (2.5–7.5%), and liquid/solid ratio (4/1–6/1), on yield, visibility, and holocellulose, a-cellulose, and lignin contents of the pulps were measured. Pulps with the highest possible holocellulose content (78.9%), cellulose content (61.8%) were obtained, and viscosity (426.8 mL/g), as well as the lowest possible lignin content (19.1%), involves the use of high ethylene glycol and soda ash (15% and 7.5%, respectively), a long cooking time (90 min), and a low liquid-to-solid ratio (4/1). A high temperature (195 °C) is also required to maximize holocellulose and cellulose content and viscosity, and a temperature of 175 °C to minimize that of lignin. On the other hand, the maximum yield (59.4%) is obtained using a long cooking time (90 min) and low values of the other operating variables. The use of intermediate operating conditions (i.e., a temperature of 187.5 °C, an ethylene glycol concentration of 15.0%, a glycol concentration of 15.0%, a soda ash concentration of 7.5%, a liquid/solid ratio of 4/1, and a cooking time of 30 min) leads to values of yield of holocellulose content, α-cellulose content, lignin content, and viscosity (52.2%, 76.5%, 58.8%, 20.40%, and 352.7 mL/g, respectively) that differ by only 12.1%, 3.0%, 4.9%, 6.8%, and 17.4% from their respective optimal values, all with significant savings in energy consumption and capital tied up in business operations. The strength-related properties of this pulp can be important with appropriate refining treatment [118]. The influence of the independent variables in olive wood cuttings pulping [i.e., cooking temperature (165–195 °C) and time (30–90 min), ethanolamine concentration (5–15%), soda concentration (2.5–7.5%), and liquid-to-solid ratio (4–6)] on pulp yield and break length, burst index, and tear index of the resulting paper sheets was further analyzed. Obtaining paper sheets with acceptable strength while saving on immobilized capital through the use of smaller structures and fewer chemical reagents involves the use of an average concentration of soda ash and low values of all other variables. Thus, the yield is 22.2% lower, and the break length, burst index, and tear index were 6.2, 29.1, and 29.6%, respectively, lower than their optimum values. It was seen that the strength properties increased when the pulp was properly refined [119]. The black liquor produced during kraft pulping was characterized. The following variables, liquid to wood ratio, active alkali concentration, sulfur content, time, and temperature on the various properties of black liquor (ph, chemical oxygen demand, total carbon, and total organic carbon) were analyzed. The least polluting black liquors are obtained by using a medium to high liquid/wood ratio, as well as a low concentration of active alkali (5%), sulfurity (8%), and temperature (137 °C) and a short time (30 min). However, the pulp and paper sheets obtained from are of poor quality; to ensure acceptable quality in the pulp and paper sheets, a high liquor/wood ratio and sulfidity (24%) should be used, as well as a medium active alkali concentration (7.5%) and temperature (165 °C) and short time (30 min) [120]. Pulp obtained from kraft pulping olive wood was used to produce sheets of paper. The experimental design adopted examined the influence of the pulping variables (temperature, time, concentration of active alkali, sulfidity, and bath/wood), and the number of PFI beating turns to which the pulp was subjected on the Shopper ± Riegler index of the pulp and on the breaking length, elongation, burst index, and tear index of the resulting sheets of paper. Obtaining paper sheets of acceptable quality involved the use of a high pulping temperature (193 °C), a medium-long pulping time (64 ± 90 min), a high concentration of active alkali (10%), high sulfidity (24%), and a large number of PFI beating revolutions (3500). To save energy and reduce capital costs, a medium-high pulping temperature (179 °C) and a short pulping time (30 min) would result in paper sheets with properties only 5 ± 20% worse than those obtained under optimal conditions [121]. Paper sheets from olive wood pulp obtained from soda, sulfite, or kraft pulp were studied to examine the influence of beating the pulp on the properties of the paper sheets. Paper sheets obtained from kraft pulp and sulfite pulp showed the highest strength, and sulfite pulp had the highest brightness. The soda pulp required more intense beating than the kraft or sulfite pulps; in fact, the PFI beater had to be operated with 40 ± 50% more revolutions per beating revolutions to obtain soda pulp with 70 ± 80° SR. With similar beating, kraft and sulfite pulps from the wood of olive provide sheets of paper with greater breaking length, elongation, burst index, and tear index than soda pulp obtained from the same wood of soda pulp from the same material, 20 ± 30%, 30 ± 50%, 50 ± 60%, and 15 ± 35% higher than those of sheets obtained from soda pulp, respectively. The content of holocellulose per unit yield of pulps from olive wood subjected to the sulfite and kraft processes exceeds that of soda pulp by 11.4 and 16.3%, respectively. The a-cell/yield ratio of sulfite pulps and kraft pulps is 11.9 and 17.2% higher than that of soda pulp, respectively. On the other hand, the lignin/yield ratio of kraft and sulfite pulps is 10.4 and 27.1% lower than that of soda pulp. All these results in higher sheet strength from kraft and sulfite pulps and higher brightness in sulfite pulp [122]. The influence of the operating conditions (active alkali concentration, temperature, and time) used in the production of wood kraft pulp from olive tree pruning residues on the yield, elongation index, burst index, and tear index of the paper sheets obtained from it as well as that of the concentration of chlorine dioxide used to bleach the pulp according to the sequence D1ED2 (alkaline-extraction-chlorine dioxide) on the yield, brightness, and viscosity of the pulp bleached pulp, was studied. Based on the pulping results, obtaining a readily bleached pulp with acceptable strength properties involves using an active alkali concentration of 25%, a temperature of 175 °C, and a time of 93 min. Based on the bleaching results, using chlorine dioxide concentrations of 7.5 and 1% in steps D1 and D2, respectively, ensures obtaining acceptably bright paste (88%) with little loss of yield (2%) and viscosity (3%). Taking into account that other varieties of olives in the Mediterranean area possess holocellulose, alpha-cellulose, and lignin content similar to that of the variety studied (picual), the pulping and bleaching conditions described above should be valid for different varieties of olive wood [123]. The effect of the operating conditions used in bleaching with hydrogen peroxide (concentration 1–5% and process time 30–210 min) and with sodium perborate (concentration 1–5%, hydrogen peroxide 0–2%, and process time 60–180 min) of olive wood waste pulp on the yield, kappa index, and viscosity of the resulting pulp and on the strength properties of the paper sheets (elongation index and burst index) in order to determine the optimal bleaching conditions for this pulp was studied. Low to medium concentrations of hydrogen peroxide (1–3%) and a high operating time (210 min) were desired in bleaching the pulp. High concentrations of sodium perborate and hydrogen peroxide (5% and 2%, respectively) and a low-to-medium operating time (60–120 min) were desired for sodium perborate bleaching. A comparison of the two bleaching agents, under similar or optimal operating conditions, revealed that sodium perborate bleaching produced lower brightness, higher viscosity than hydrogen peroxide bleaching. Besides, both provided similar elongation index and burst index values for sodium perborate bleaching compared to hydrogen peroxide bleaching [124]. The influence of the operating conditions used in the bleaching of pulp from olive wood residues (i.e., hydrogen peroxide concentration and time) on the yield, viscosity of the resulting pulp, and strength-related properties of the paper sheets was studied to determine the optimal bleaching conditions for this pulp. Pulps bleached with hydrogen peroxide at different sequences (oxygen, ozone, chlorine dioxide, and alkaline extractions) were paralleled. Hydrogen peroxide bleaching was found to be suitable for this paste. Significant improvements in viscosity were obtained compared to other bleaching sequences such as oxygen, ozone, and chlorine dioxide. Hydrogen peroxide bleaching decreased the kappa index* by 51.3% compared to ozone bleaching, 25.0% compared to chlorine dioxide (D), and 6.3% less than combined chlorine-alkali dioxide (DE) extraction. To achieve kappa indices of 50.9% and 37.9% lower than the index achieved by hydrogen peroxide, oxygen (LaOp) and ozone (LaO(LaZ)R) sequences were required, respectively. Low to medium levels of hydrogen peroxide concentrations (1–3%) and high reaction times (210 min) proved to be suitable for bleaching olive cut residues into pulp. This approach may be used on this residue to supply adequately bleached pulp. This approach may be used on this residue to supply adequately bleached pulp. Hydrogen peroxide bleaching was suitable for cellulose losing pulp from the olive trimming residue. A great improvement in pulp viscosity was obtained compared to other bleaching sequences such as oxygen ozone and chlorine dioxide. Low to medium levels hydrogen peroxide concentrations and reaction times are sufficient for bleaching pulp cellulose trimming residues [125].

## 3. Materials and Methods

### 3.1. Search Strategy

Basing on Preferred Reporting Items for Systematic Reviews and Meta-Analyses (PRISMA) guidelines, a thorough systematic literature search was performed in December 2020 and comprised all reports published to date. The search was carried out on PubMed and Scopus specialized databases by using different combinations of two keywords: olive bark, or olive wood, or olive roots, or olive pruning and “secondary metabolites”, distribution, chemistry, “antioxidant activity”, antioxidant, inflammation, “antiobesity activity”, obesity, “antiglicemic activity”, “antiatherosclerotic activity”, “metabolic syndrome”, “antihypertensive activity” “pharmacological properties”, “molecular targets”, “anticancer activity”, cancer, tumor, cytotoxic, cytotoxicity, apoptosis, “cell cycle arrest”, chemosensitizing, bio-composite, “dendrometric parameters”, “mechanical properties”, furniture, design, “indoor application”, buildings, fibers, biorefinery, pellets, compost. We required full-text for investigation; however, if not available and we did not try to find unpublished data.

### 3.2. Study Selection

Literature selection was made according to the inclusion criteria: only olive parts included in the keywords, only articles published in English and containing keywords in the title or in the abstract were included. Other review articles, editorials, book chapters, conference papers, surveys, letters, notes, manuscripts without full text available, or articles that did not fit the inclusion criteria were not included in this systematic review. The study selection was performed by three independent investigators (R.B., V.L.G., F.L., and M.P.) by a first screening of the articles basing on the title and abstract and then analyzing the full-texts. In cases of non-consensus, authors tried to resolve any disagreements by discussion or, if necessary, two more independent reviewers were consulted (L.M. and L.T.). The selected articles were carefully reviewed to identify or exclude the manuscripts that did not fit the criteria described above. Additional papers were added in this review after the analysis of the bibliography from the included articles.

### 3.3. Data Extraction

Data were collected and analyzed by the authors to extract information on olive by-products, as well as experimental design, cellular and animal models, and major outcomes on general mechanisms involved in biological activities and industrial applications.

### 3.4. Methodological Quality Assessment

The methodological quality and the risk of bias assessment were carried out independently by the authors, using a checklist adapted from *Cochrane Handbook for Systematic Reviews of Interventions*, appropriately adjusted for animal intervention study (SYRCLE’s) [17,126] and clinical trials [127]. For the environmental study, the quality assessment was based on Collaboration for Environmental Evidence (CEE) Guidelines and Standards for Evidence Synthesis in Environmental Management, in conformance to ROSES reporting standards [18,19]. The appraisal of the methodological quality of the selected studies was based on the presence or absence of information regarding the main objectives and findings, randomization of the treatment allocation, sampling, blinded drug administration, blinded outcome assessment and outcome measurements, method description, number of replicates, as reported in Table 5, Table 6 and Table 7. Only studies that reported a positive rating in all considered parameters were considered of higher methodological quality [128]. Conversely, the studies that did not completely fulfil the criteria were included in the medium risk of bias, while those that completely lacked this information were deemed at high risk of bias.

## 4. Conclusions

As it is possible to deduce from this systematic review, olive trees by-products represented an important resource for either agroindustrial employment or healthy properties. Data regarding the potential biological activity of extracts from olive roots, wood, bark, and pruning were analyzed. It was seen that olive trees by-products are rich in molecules with antioxidant, antimicrobial, cardioprotective, and anticancer activity, representing a promising candidate for treating several human diseases. Furthermore, they were strongly analyzed as pulp raw material under different processes using organic solvents. Olive tree branches have also been proven to have a good nutritional value for the rationing and formulation of diets for animals. Besides, it is noteworthy that research extended to the concept of a biorefinery based on olive tree pruning residues because it has been shown to contain compounds (e.g., oligosaccharides) suitable for feed, food, pharmaceutical, cosmetics, or agrochemistry industrial sectors. In conclusion, this systematic review represents a summary of the many potentialities of olive trees by-products analyzedin studies reported in the literature. It can constitute a start-point for new studies giving a new life to products otherwise destined to be waste only.

## Figures and Tables

**Table 1:** Bioactive specialized metabolites identified in bark, root, wood, and pruning of *O. europaea*.

**Secoiridoids**			
**Compound**	**Formula**	**Structure**	**Part of *O. europaea***
Elenolic acid glucoside isomer	C_17_H_25_O_11_	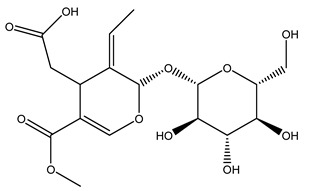	Bark [1]
Fraxamoside	C_25_H_30_O_13_	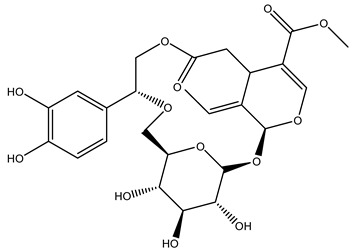	Wood [2,3]
Isojaspolyoside A	C_42_H_54_O_23_	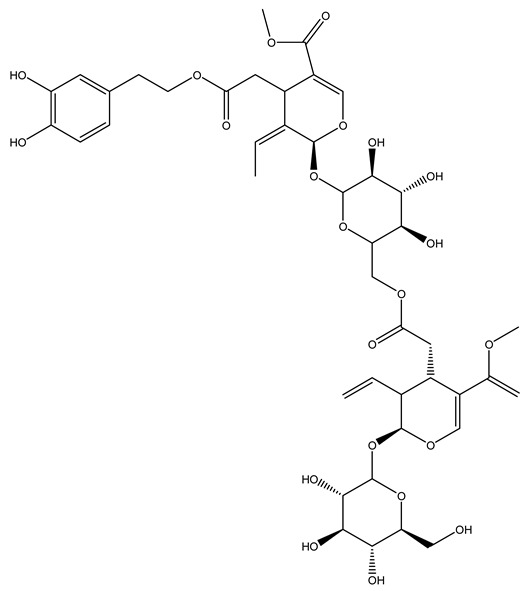	Wood [4,5,6]
Jaspolyoside	C_42_H_54_O_23_	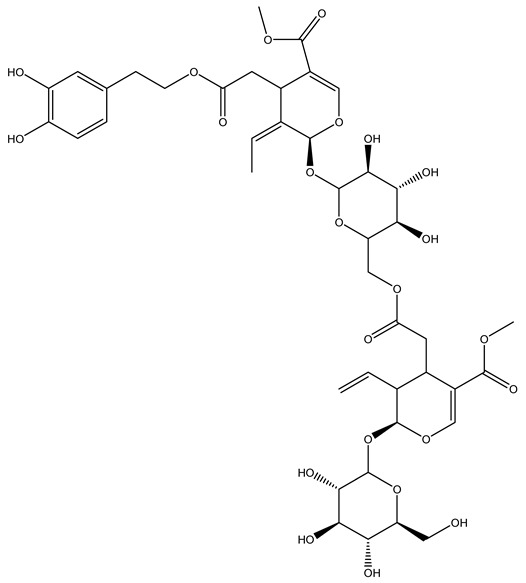	Wood [3,4,5,6]
Jaspolyanoside	C_43_H_64_O_13_	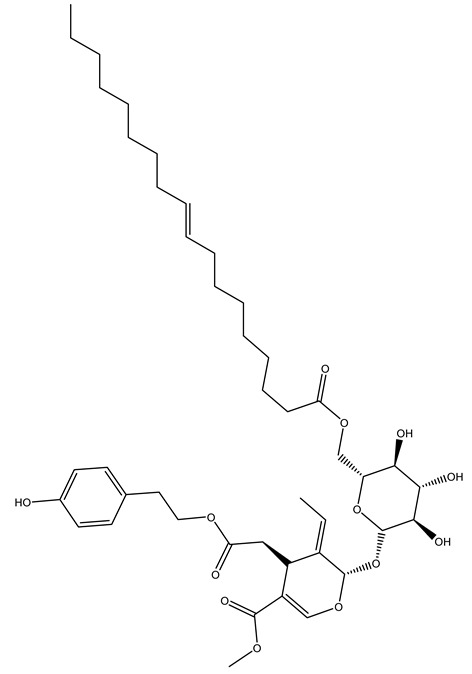	Wood [4,5,6]
Lucidumoside C	C_27_H_36_O_14_	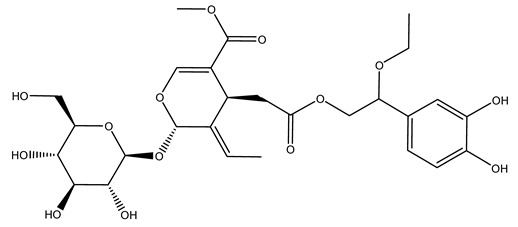	Wood [2,3]
Ligustroside	C_25_H_32_O_12_	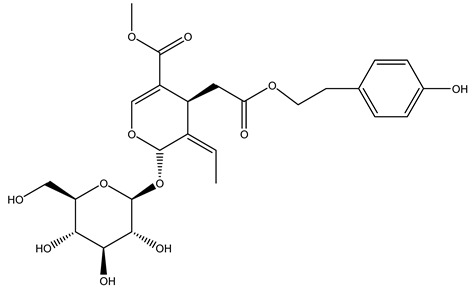	Bark [1] Wood [2,3,4,5,6,7]
Ligustroside 3′-*O*-*β*-d-glucoside	C_25_H_32_O_12_	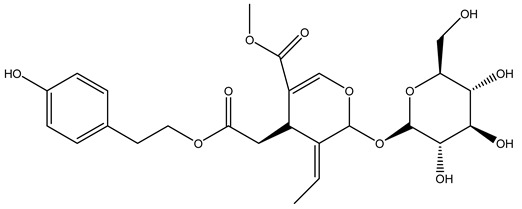	Wood [4,5,6,7]
Nuzhenide	C_31_H_42_O_17_	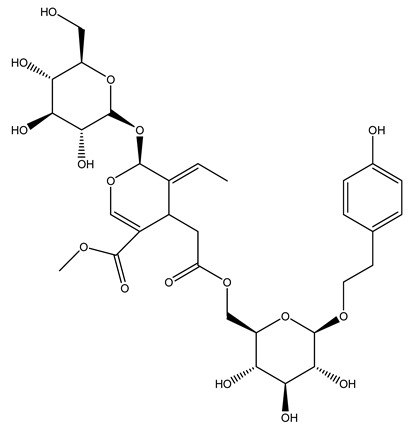	Bark [1]
Oleoside	C_16_H_22_O_11_	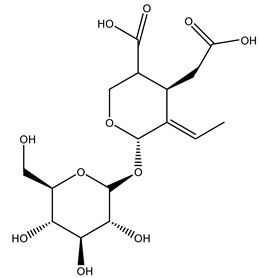	Bark [1] Wood [3]
Oleoside 11-methyl ester	C_17_H_24_O_11_	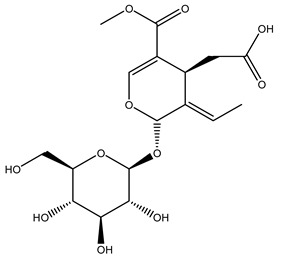	Wood [3,6]
Oleuropein	C_25_H_32_O_13_	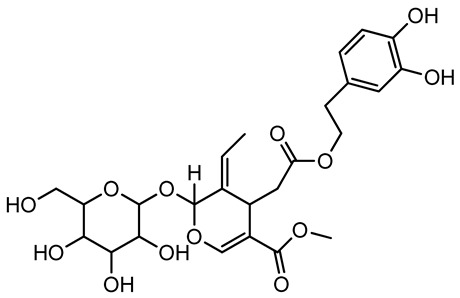	Bark [1,8] Root [9] Wood [2,3,4,5,6,7,10,11] Pruning [12]
Oleuropein 3′-*O*-*β*-d-glucoside	C_25_H_32_O_13_	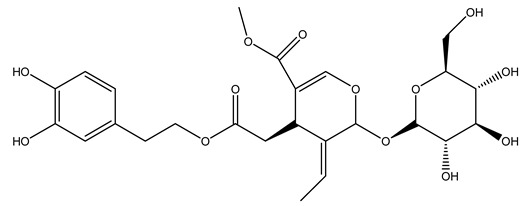	Wood [4,5]
Oleuropein aglycone	C_19_H_22_O_8_	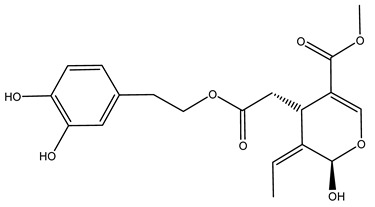	Wood [3]
Oleuropein diglucoside isomers	C_31_H_42_O_18_	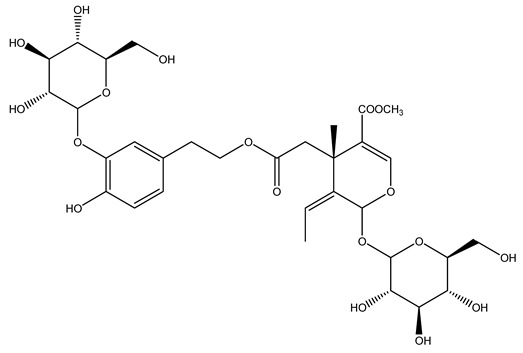	Bark [1]
Demethyl oleuropein	C_24_H_30_O_13_	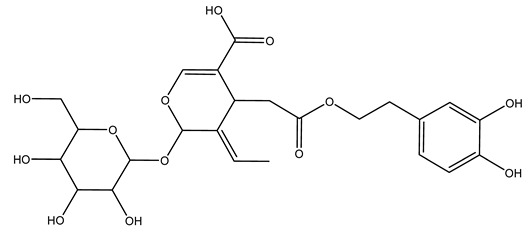	Wood [3]
2″-Hydroxy oleuropein	C_25_H_32_O_14_	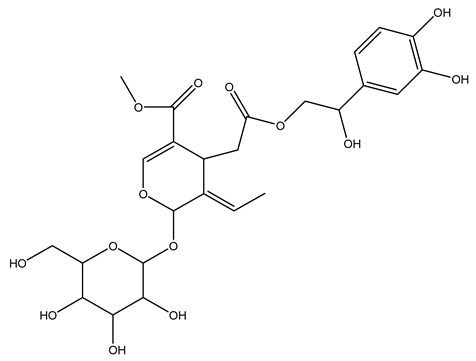	Wood [3,5,6]
7″*S*-Hydroxy oleuropein	C_25_H_32_O_14_	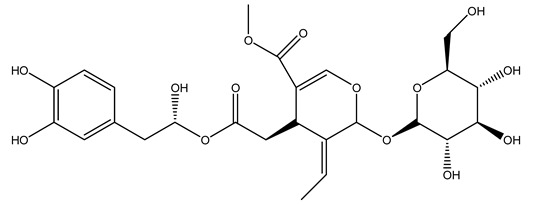	Wood [3,4,5,7]
10-Hydroxy oleuropein	C_25_H_32_O_14_	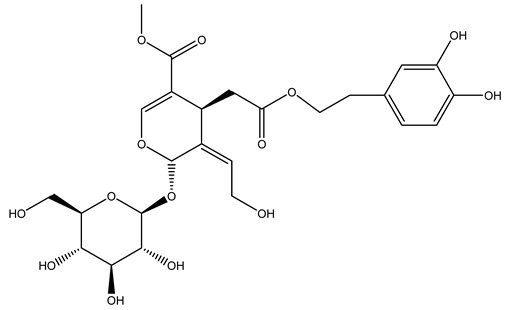	Bark [1] Wood [3] Pruning [13]
(7″*R*)-7″-Ethoxy oleuropein (or (7″*S*)-7″-Ethoxy oleuropein)	C_26_H_34_O_12_	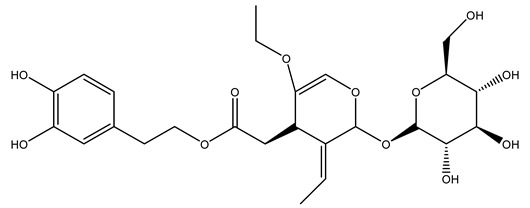	Wood [5,7]
3″-Methyl ether oleuropein	C_26_H_34_O_13_	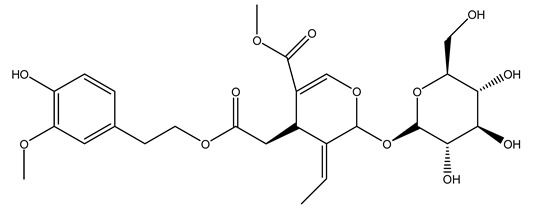	Wood [4,5]
Dihydro oleuropein	C_25_H_36_O_13_	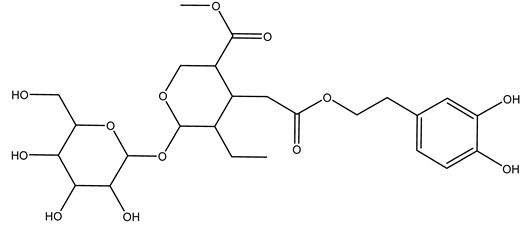	Wood [2,3]
Methoxy oleuropein	C_26_H_34_O_14_	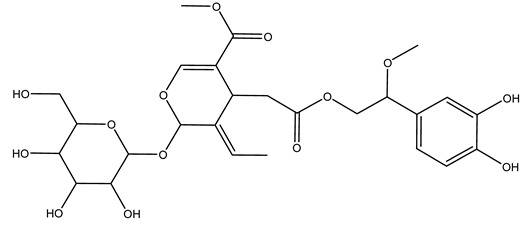	Bark [1] Wood [2,3]
Secologanoside	C_16_H_22_O_11_	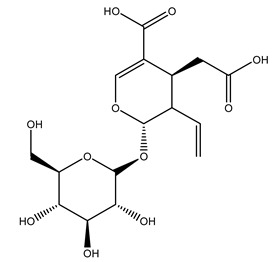	Bark [1] Wood [3]
**Iridoid**			
**Compound**	**Formula**	**Structure**	**Part of *O. europaea***
7-Deoxy loganic acid	C_16_H_24_O_9_	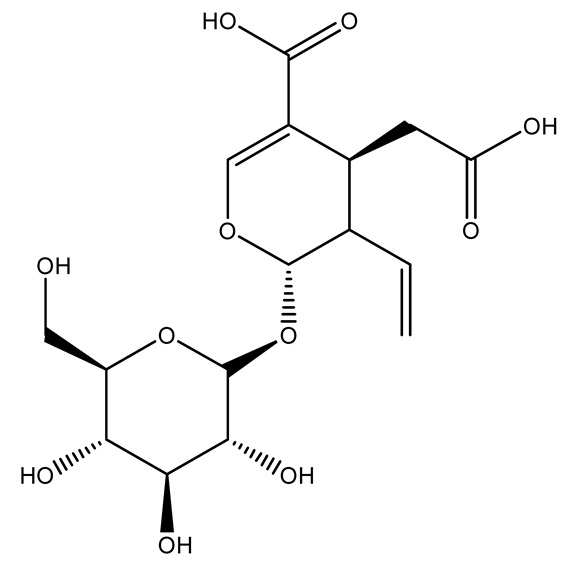	Wood [2,3,4,5,6]
Loganin	C_17_H_26_O_10_	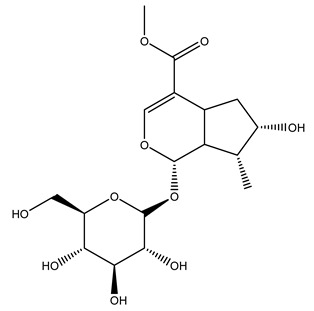	Wood [3] Pruning [13]
**Phenolic Compounds and Derivatives**			
**Compound**	**Formula**	**Structure**	**Part of *O. europaea***
Acetophenone, 3,5-dimetoxy-4-hydroxy	C_10_H_12_O_4_	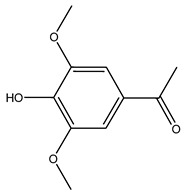	Pruning [14]
Acetovanillone	C_9_H_10_O_3_	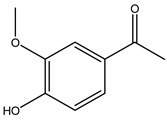	Pruning [14]
Benzaldehyde	C_7_H_6_O	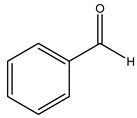	Pruning [13,15,17]
Benzaldehyde, *p*-carbomethoxy	C_9_H_8_O_3_	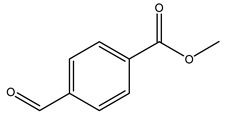	Pruning [12]
Benzaldehyde, 3,4-dihydroxy	C_7_H_6_O_3_	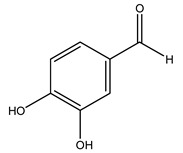	Pruning [12]
Benzene, 1,4-dimethoxy	C_6_H_4_(OCH_3_)_2_	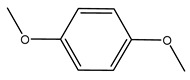	Pruning [16]
Benzene, 4-methyl-1,2-dihydroxy	C_7_H_8_O_2_	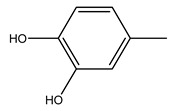	Pruning [16]
1,2-Benzenediol	C_6_H_6_O_2_	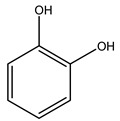	Pruning [16]
1,3-Benzenediol, 4-ethyl	C_8_H_10_O_2_	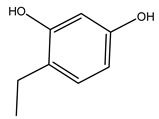	Pruning [13]
Benzoic acid	C_7_H_6_O_2_	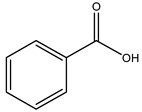	Wood [10,11]
Benzoic acid, 2-formyl-methyl ester	C_9_H_8_O_3_	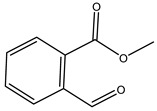	Pruning [13]
Benzyl alcohol	C_7_H_8_O	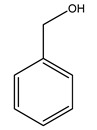	Pruning [12]
Caffeic acid	C_9_H_8_O_4_	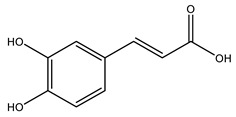	Bark [1,8] Wood [3]
Catechol	C_6_H_6_O_2_	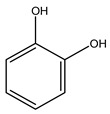	Pruning [13,14]
Catechol, 4-methyl	C_7_H_8_O_2_	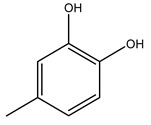	Pruning [14] Wood [17]
*o*-Cresol	C_7_H_8_O	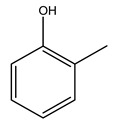	Pruning [14]
*m*-Cresol	C_7_H_8_O	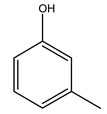	Pruning [13,14]
*p*-Cresol	C_7_H_8_O	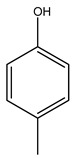	Pruning [13,14]
*p*-Coumaric acid	C_9_H_8_O_3_	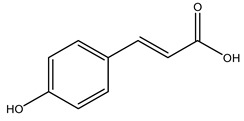	Bark [8] Wood [10,11]
Ethanone, 1-(4-hydroxy-3-metoxyphenyl)	C_9_H_10_O_3_	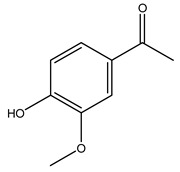	Pruning [13]
Eugenol	C_10_H_12_O_2_	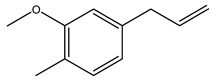	Pruning [16]
Eugenol, methoxy	C_11_H_14_O_3_	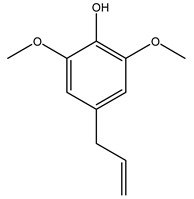	Pruning [12]
Ferulic acid	C_10_H_10_O_4_	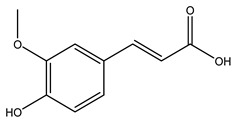	Bark [1,8] Pruning [14]
Formic acid phenyl ester	C_7_H_6_O_2_	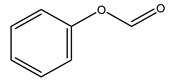	Pruning [13]
5-Formylsalicyil aldehyde	C_8_H_6_O_3_	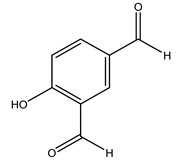	Pruning [13]
*o*-Guaiacol	C_7_H_8_O_2_	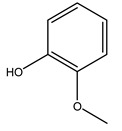	Pruning [13,14,15,16]
Guaiacyl acetone	C_10_ H_12_ O_3_	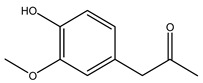	Pruning [12]
Homovanillyl alcohol	C_9_H_12_O_3_	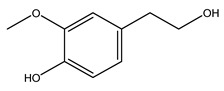	Bark [1] Wood [3] Pruning [12,15]
*p*-Hydroxy benzoic acid	C_7_H_6_O_3_	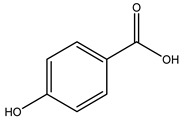	Bark [1] Wood [3]
Hydroxy tyrosol	C_8_H_10_O_3_	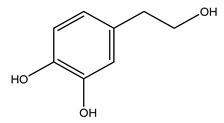	Bark [1,8] Root [9] Wood [2,3,4,5,6,10,11] Pruning [12]
Hydroxy tyrosol-4-glucoside	C_13_H_18_O_8_	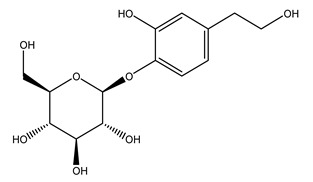	Bark [1] Wood [3]
Isoeugenol	C_10_H_12_O_2_	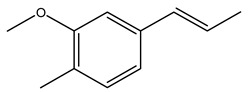	Pruning [16]
2-Pentanone, 1(-2,4,6-tryhydroxy phenyl)	C_11_H_14_O_4_	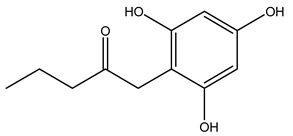	Pruning [12]
Phenylacetic acid, *p*-hydroxy	C_8_H_8_O_3_	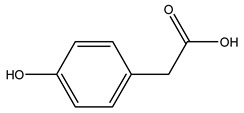	Bark [8]
2-Phenylethyl alcohol	C_8_H_10_O	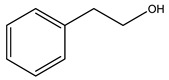	Pruning [12]
Phenol	C_6_H_6_O	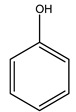	Pruning [13,14,16]
Phenol, 2-methyl	C_7_H_8_O	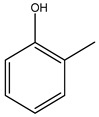	Pruning [16]
Phenol, 2,4-di-*tert*-butyl	C_14_H_22_O	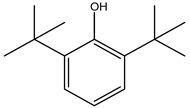	Pruning [18]
Phenol, 2,6-dimethoxy	C_8_H_10_O_3_	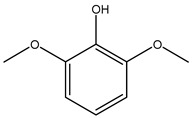	Pruning [16]
Phenol, 2,6-dimethoxy-4-(2-propeny-)	C_11_H_14_O_3_	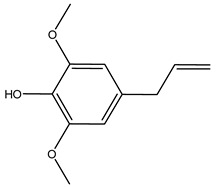	Pruning [13]
Phenol-4-ethyl	C_8_H_10_O	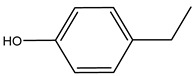	Pruning [13]
Phenol, 4-ethyl-2-methoxy	C_9_H_12_O_2_	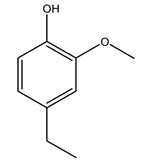	Pruning [16]
Phenol, 4-methoxy	C_7_H_8_O_2_	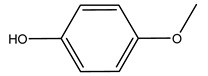	Pruning [12]
Phenol, 2-methoxy-4-(1-propenyl)-*E*	C_10_H_12_O_2_	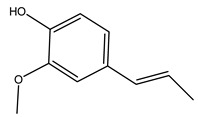	Pruning [13]
Phthalate, diethyl	C_12_H_14_O_4_	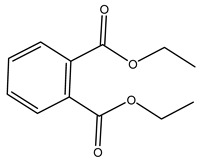	Pruning [13]
Protocatechic acid	C_7_H_6_O_4_	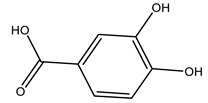	Wood [10,11]
Quinic acid	C_7_H_12_O_6_	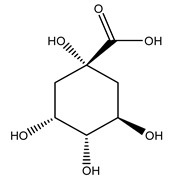	Wood [3]
Syring aldehyde	C_9_H_10_O_4_	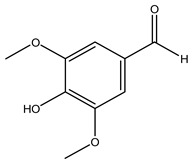	Pruning [12,14,15]
Syringic acid	C_9_H_10_O_5_	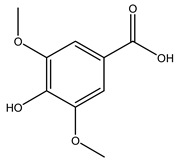	Pruning [14]
Syringol	C_8_H_10_O_3_	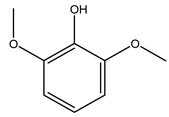	Pruning [13,14,15]
Toluene	C_7_H_8_	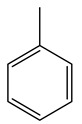	Pruning [16]
1,2,4-Trimethoxy benzene	C_9_H_12_O_3_	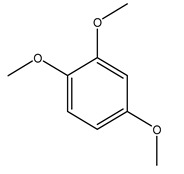	Pruning [16]
1,2,3-Trimethoxy-5-methylbenzene	C_10_H_14_O_3_	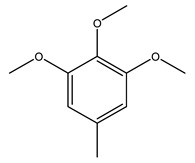	Pruning [16]
3,4,5-Trimethoxy phenylacetic acid	C_11_H_14_O_5_	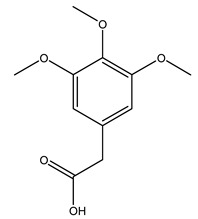	Pruning [12]
Tyrosol	C_8_H_10_O_2_	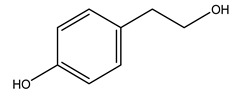	Bark [8] Root [9] Wood [2,4,5,6,10,11] Pruning [12]
Vanillic acid	C_8_H_8_O_4_	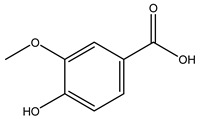	Bark [8] Pruning [12,14]
Vanillin	C_8_H_8_O_3_	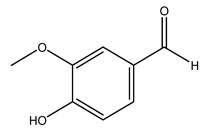	Bark [1,8] Wood [3,10,11] Pruning [12,14]
*p*-Vinylguaiacol	C_9_H_10_O_2_	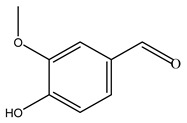	Pruning [13]
*o*-Xylene	C_8_H_10_	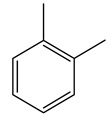	Pruning [13]
**Flavonoids**			
**Compound**	**Formula**	**Structure**	**Part of *O. europaea***
Apigenin *O*-rutinoside	C_27_H_30_O_14_	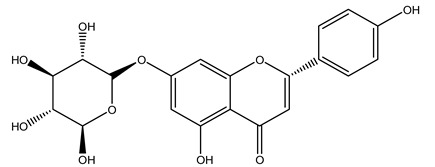	Pruning [13]
Catechin	C_15_H_14_O_6_	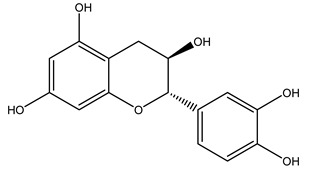	Wood [11]
Chrysoeriol-7-*O*-glucoside	C_22_H_22_O_11_	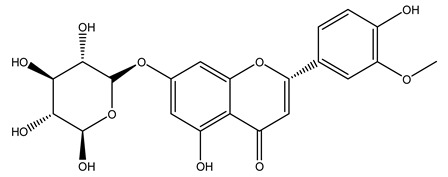	Pruning [13]
Eriodyctiol	C_15_H_12_O_6_	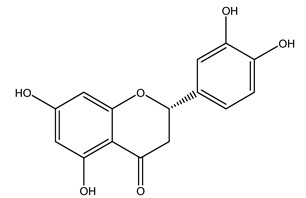	Bark [1] Wood [6]
Fustin	C_15_H_12_O_6_	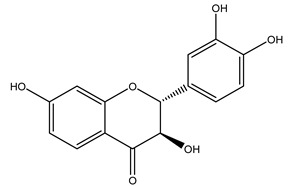	Bark [1]
Kaempferol	C_15_H_10_O_6_	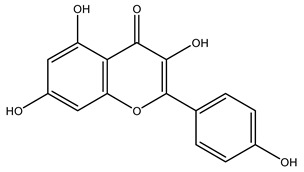	Wood [17]
Luteolin	C_15_H_10_O_6_	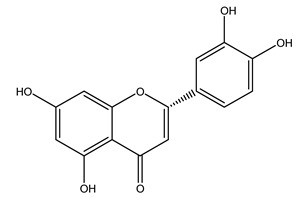	Bark [1]
Luteolin glucoside	C_21_H_20_O_11_	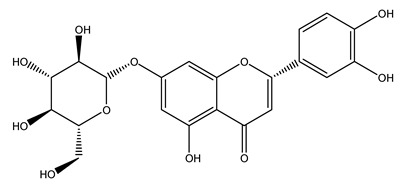	Bark [1] Pruning [13]
Luteolin 4-*O*-*β*-d-glucopyranoside	C_21_H_20_O_11_	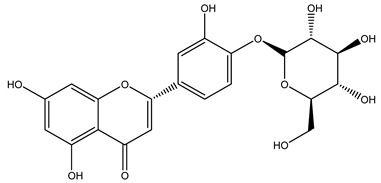	Pruning [13]
Luteolin diglucoside	C_27_H_30_O_16_	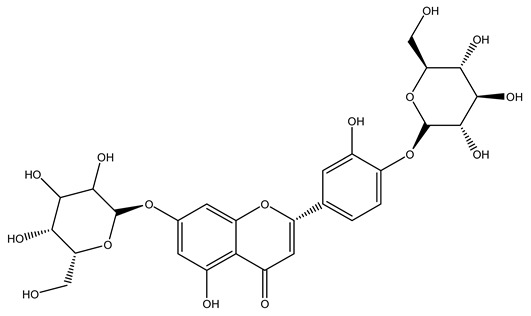	Pruning [13]
Naringenin	C_15_H_12_O_5_	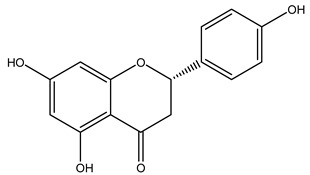	Bark [1]
Naringenin-hexoside	C_21_H_22_O_10_	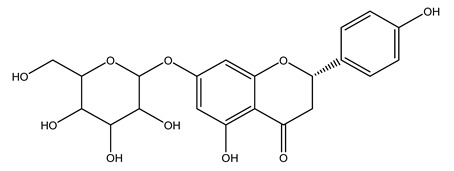	Bark [1]
Quercetin	C_15_H_10_O_7_	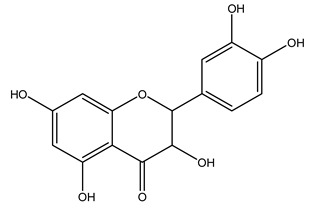	Bark [1]
Rutin	C_27_H_30_O_16_	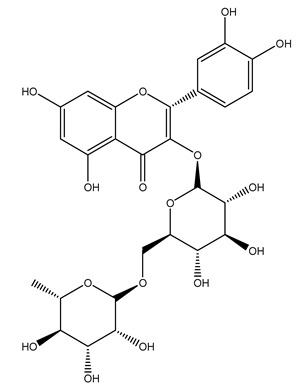	Pruning [13]
Taxifolin	C_15_H_12_O_7_	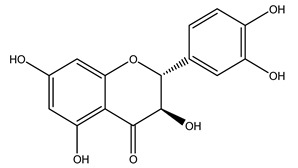	Bark [1] Pruning [13]
**Cinnamic Acid and Derivatives**			
**Compound**	**Formula**	**Structure**	**Part of *O. europaea***
Cinnamaldehyde, 4-hydroxy-2-methoxy	C_10_H_10_O_3_	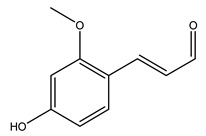	Pruning [12]
Forsythiaside (Acteoside isomer)	C_29_H_36_O_15_	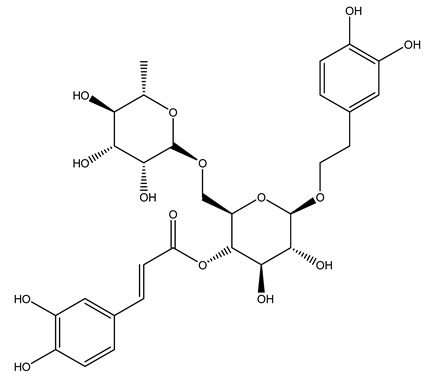	Bark [1]
Isoacteoside (Acteoside isomer)	C_29_H_36_O_15_	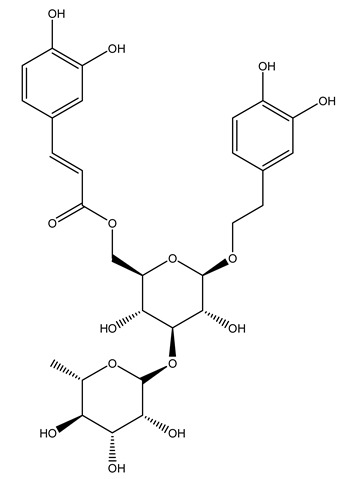	Bark [1]
Isoverbascoside	C_29_H_36_O_15_	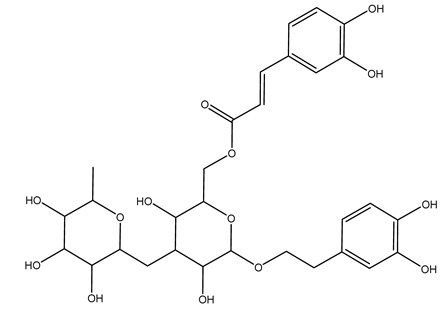	Wood [3]
Verbascoside (syn. Acteoside or Decaffeoylverbascoside)	C_29_H_36_O_15_	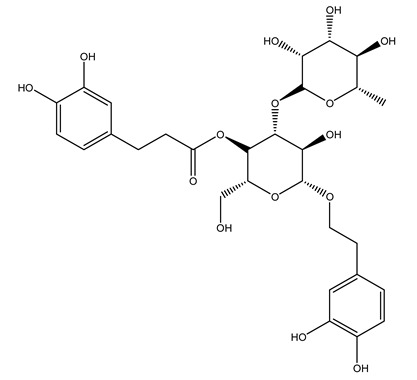	Bark [1] Wood [3]
Verbascoside, *β*-hydroxy	C_29_H_36_O_15_	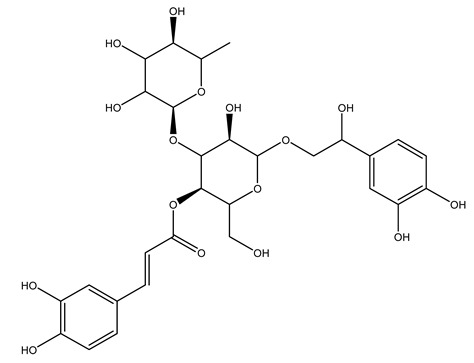	Wood [3]
**Hydroxycoumarin Derivatives**			
**Compound**	**Formula**	**Structure**	**Part of *O. europaea***
Aesculetin (syn. Dihydroxycoumarin)	C_9_H_6_O_4_	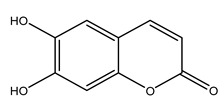	Wood [3]
Cichoriin	C_15_H_16_O_9_	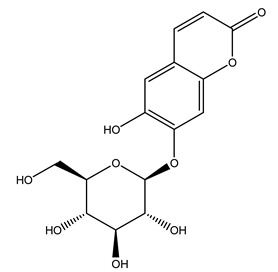	Bark [1]
Dimeresculetin	C_18_H_10_O_8_	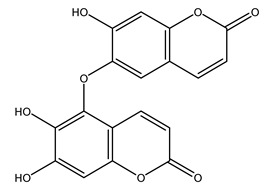	Bark [1]
Esculetin	C_9_H_6_O_4_	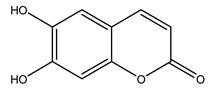	Bark [1]
Esculetin, methyl	C_10_H_8_O_4_	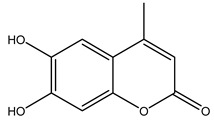	Bark [1]
Esculin (syn. Aesculin and Esculetin hexoside)	C_15_H_16_O_9_	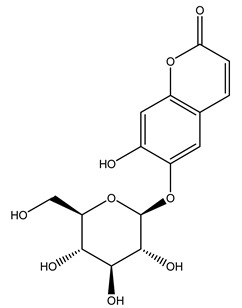	Bark [1] Wood [3]
Scopoletin	C_10_H_8_O_4_	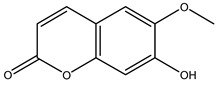	Bark [1] Wood [3]
**Furans**			
**Compound**	**Formula**	**Structure**	**Part of *O. europaea***
Furan, 2-acetyl	C_6_H_6_O_2_	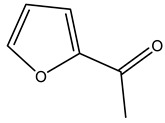	Pruning [12]
Furan, 2-acetyl-5-methyl	C_7_H_8_O_2_	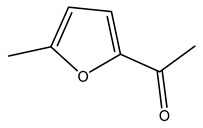	Pruning [12]
Furan, 2,5-dimethyl	C_6_H_8_O	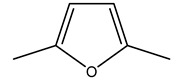	Pruning [13]
2-Furan methanol	C_5_H_6_O_2_	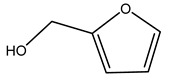	Pruning [13,15,16]
2-Furan-5-methyl carboxaldehyde	C_6_H_6_O_2_	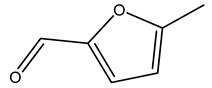	Pruning [16]
2-Furan methanol, Tetrahydro	C_5_H_10_O_2_	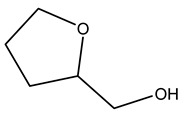	Pruning [16]
2,5-Furandione, 3-methyl	C_5_H_4_O_3_	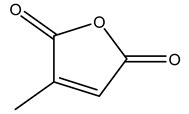	Pruning [15]
2,5-Furandione, 3,4-dimethyl	C_6_H_6_O_3_	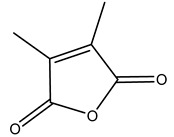	Pruning [12]
2-Furanone, 2,5-dihydro-3,5-dimethyl	C_6_H_8_O_2_	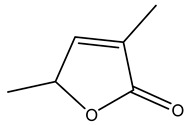	Pruning [12]
3-(2*H*)-Furanone, 2,5-dimethyl-4-hydroxy	C_6_H_8_O_3_	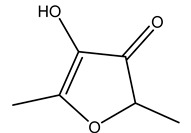	Pruning [12]
2-(3*H*)-Furanone, 5-ethoxydihydro (syn. *γ*-Ethoxybutyrolactone)	C_6_H_10_O_3_	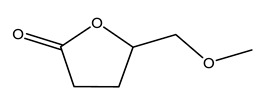	Pruning [12]
3-(2*H*)-Furanone, 2-(1-hydroxy-1-methyl-2-oxopropyl)-2,5-dimethyl	C_10_H_14_O_4_	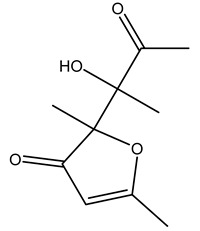	Pruning [12]
2-(3*H*)-Furanone, 5-methyl	C_5_H_6_O_2_	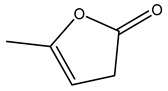	Pruning [16]
1-2-(Furanyl)-ethanone	C_6_H_6_O_2_	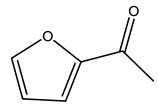	Pruning [13,15,16]
4-(2-Furanyl)-3-buten-2-one	C_8_H_8_O_2_	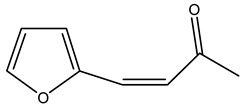	Pruning [12]
Furfural	C_5_H_4_O_2_	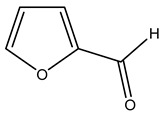	Pruning [12,13,16]
Furfural, hydroxymethyl	C_6_H_6_O_3_	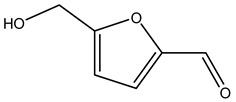	Pruning [15,19]
Furfural, 5-methyl	C_6_H_6_O_2_	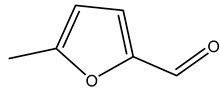	Pruning [12]
Furfuraldehyde, 5-acetoxymethyl	C_8_H_8_O_4_	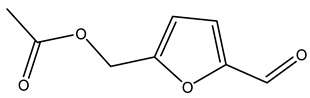	Pruning [12]
2-Furoic acid, 3-methyl	C_6_H_6_O_3_	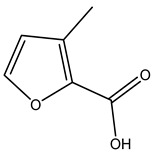	Pruning [12]
Butyrolactone	C_4_H_6_O_2_	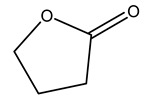	Pruning [12,16]
2,3-Dymethyl-4-hydroxy-2-butenoic acid lactone	C_6_H_8_O_2_	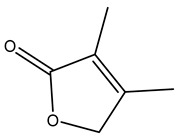	Pruning [12]
**Nitrogen Compounds**			
**Compound**	**Formula**	**Structure**	**Part of *O. europaea***
Acetylnicotinate, 5-methyl	C_8_H_9_NO_2_	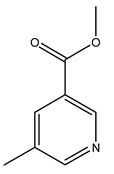	Pruning [12]
*N*-Acetyltyramine	C_10_H_13_NO_2_	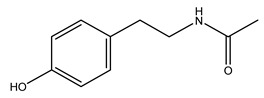	Pruning [12]
Benzoate, methyl 4-(methylamino)	C_9_H_11_NO_2_	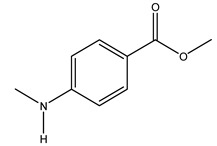	Pruning [12]
Colchifoleine	C_21_H_23_NO_7_	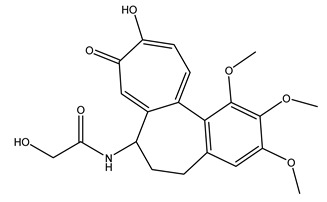	Wood [17]
d-Cycloserine	C_3_H_6_N_2_O_2_	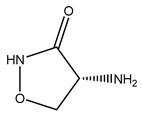	Wood [17]
Malonamic acid	C_3_H_5_NO_3_	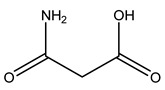	Wood [17]
Nicotinate, methyl	C_7_H_7_NO_2_	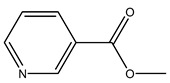	Pruning [12]
Pterin, 6-carboxy	C_7_H_5_N_5_O_3_	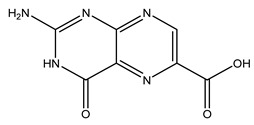	Pruning [12]
Pyridine, 2-ethenyl	C_7_H_7_N	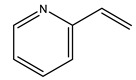	Pruning [16]
Pyridine, 4-ethenyl	C_7_H_7_N	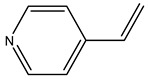	Pruning [16]
Pyridine, 3-ethyl	C_7_H_9_N	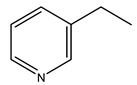	Pruning [15,16]
Pyrrole, 2-carboxaldehyde	C_5_H_5_NO	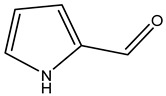	Pruning [12]
3-Vinylpyridine	C_7_H_7_N	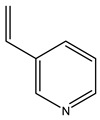	Pruning [12]
5-Vinylpyridine, 3-carbomethoxy-	C_9_H_9_NO_2_	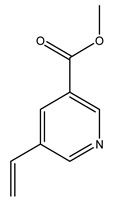	Pruning [12]
**Fatty Acids**			
**Compound**	**Formula**	**Structure**	**Part of *O. europaea***
*n*-Hexadecanoic acid	C_16_H_32_O_2_	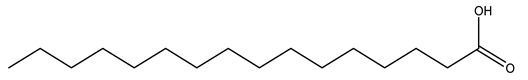	Pruning [12]
Isopropyl myristate	C_17_H_34_O_2_	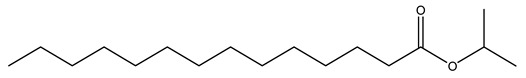	Pruning [12]
Oleic acid	C_18_H_34_O_2_	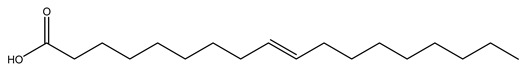	Pruning [13]
Palmitic acid	C_16_H_32_O_2_	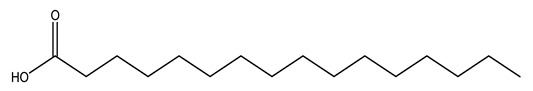	Pruning [13]
Stearic acid	C_18_H_36_O_2_	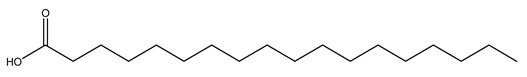	Pruning [13]
**Lignans**			
**Compound**	**Formula**	**Structure**	**Part of *O. europaea***
Cycloolivil	C_20_H_24_O_7_	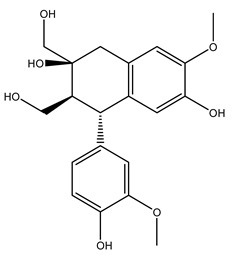	Wood [2,3,4,5]
Cycloolivil glycoside	C_26_H_34_O_12_	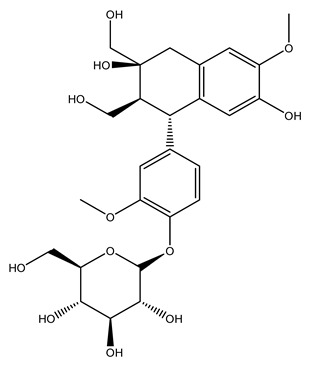	Wood [5]
(−)-Olivil	C_20_H_24_O_7_	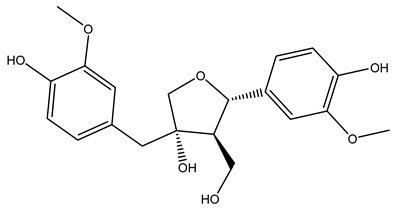	Wood [3,6] Pruning [13]
Pinoresinol-(+)-1-hydroxy-1-*O*-*β*-d-glucopyranoside	C_26_H_32_O_12_	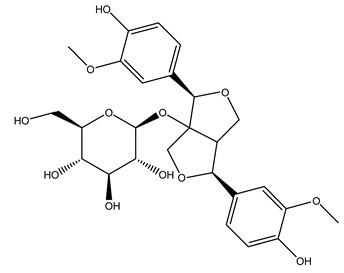	Wood [3,5,6]
(−)-Olivil 4-*O*-*β*-d-glucopyranoside	C_26_H_34_O_12_	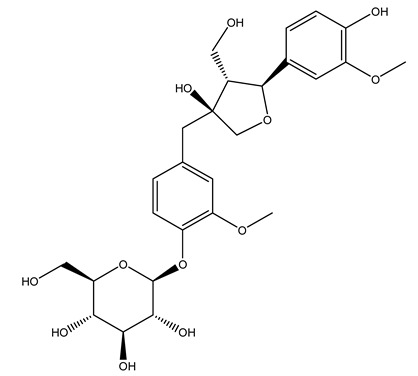	Wood [3,5,6]
**Sugars**			
**Compound**	**Formula**	**Structure**	**Part of *O. europaea***
Arabinose	C_5_H_10_O_5_	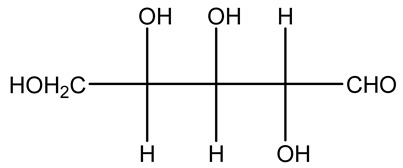	Wood [20] Pruning [14,19,21]
Fructose	C_6_H_12_O_6_	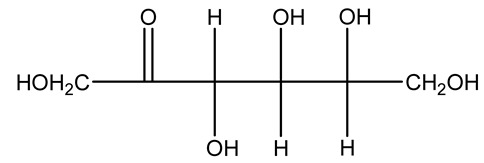	Root [22] Pruning [19]
Galactose	C_6_H_12_O_6_	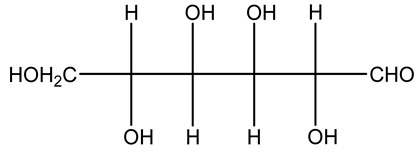	Wood [20] Pruning [19]
Glucose	C_6_H_12_O_6_	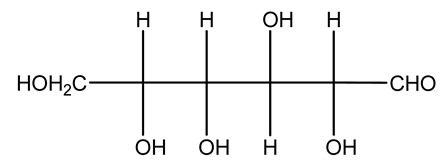	Root [22] Wood [20] Pruning [13,14,19,21,23]
Inositol	C_6_H_12_O_6_	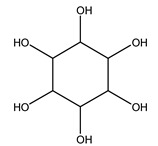	Root [9]
Mannitol	C_6_H_14_O_6_	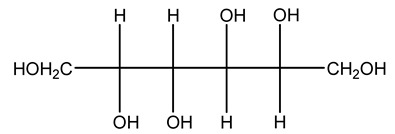	Root [9] Wood [3,5] Pruning [19]
d-Mannitol,1,4-anhydro	C_6_H_12_O_5_	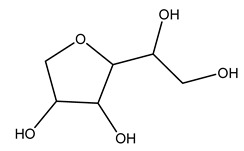	Pruning [16]
Mannose	C_6_H_12_O_6_	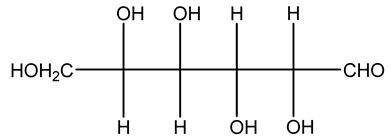	Wood [20] Pruning [19]
Sorbitol	C_6_H_14_O_6_	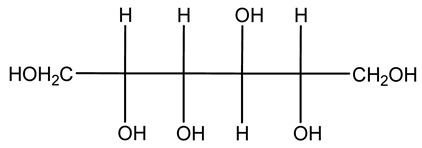	Root [9]
Stachyose	C_24_H_42_O_21_	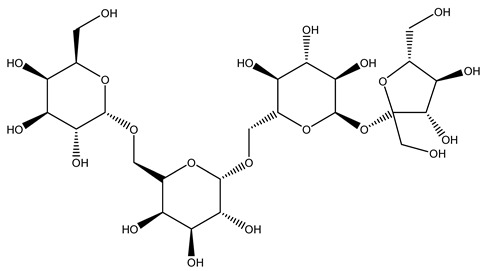	Wood [3]
Sucrose	C_12_H_22_O_11_	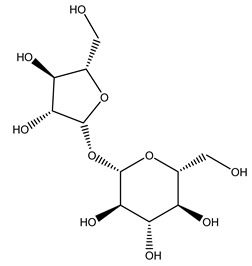	Root [22]
Verbascose	C_30_H_52_O_26_	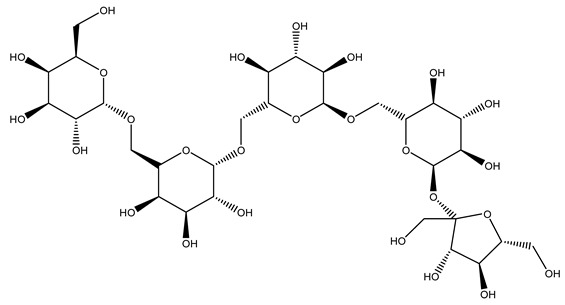	Wood [3]
Xylitol	C_5_H_12_O_5_	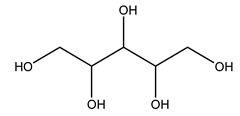	Pruning [21]
Xylose	C_5_H_10_O_5_	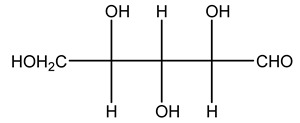	Wood [20] Pruning [13,14,19,21]
**Cellulose and Hemiellulose**			
**Compound**	**Formula**	**Structure**	**Part of *O. europaea***
Cellulose	(C_6_H_10_O_5_)_n_	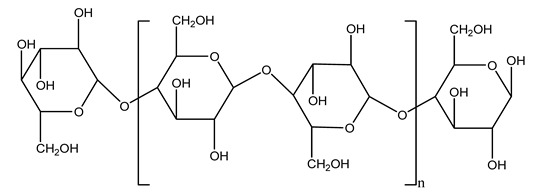	Pruning [13,24,25]
Arabinan	(C_15_H_24_O_12_)_n_	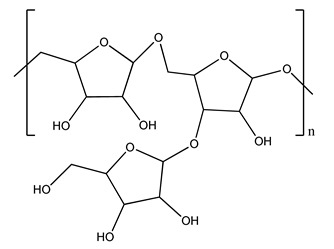	Pruning [13,25]
Galactan	C_14_H_26_O_11_	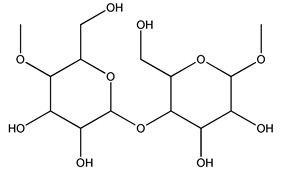	Pruning [13]
Mannan	C_24_H_42_O_21_	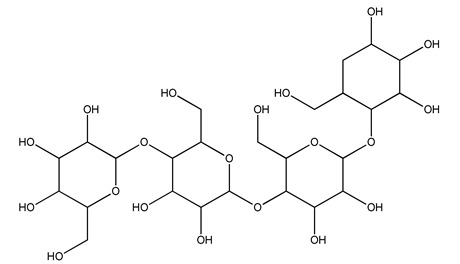	Pruning [13]
**Other Compounds**			
**Compound**	**Formula**	**Structure**	**Part of *O. europaea***
Acetaldehyde	C_2_H_4_O	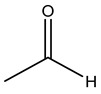	Wood [17] Pruning [16]
Acetic acid	CH_3_COOH	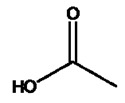	Wood [17] Pruning [12,16,17,19,21]
Acetic anhydride	C_4_H_6_O_3_	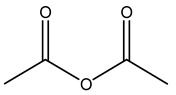	Pruning [16]
1-Acetoxyacetone	C_5_H_8_O_3_	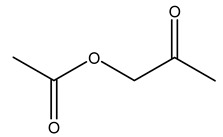	Pruning [12]
Ascorbylhexoside	C_12_H_18_O_11_	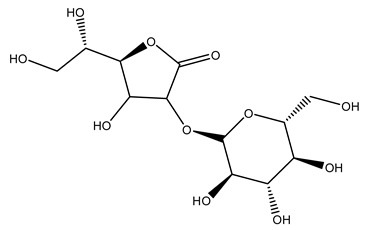	Wood [3]
2,3-Butanedione	C_4_H_6_O_2_	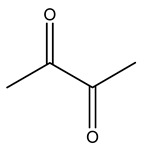	Pruning [16]
Butanoate, 2-propenyl	C_7_H_12_O_2_	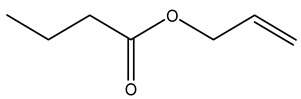	Pruning [16]
2-Butanone	C_4_H_8_O	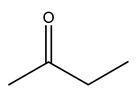	Pruning [16]
2-Butanone, 1-acetyloxy	C_6_H_10_O_3_	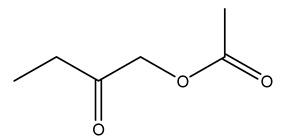	Pruning [12]
2-Butanone, 1-hydroxy	C_4_H_8_O_2_	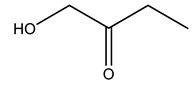	Pruning [16]
2-Butanone, 3-hydroxy	C_4_H_8_O_2_	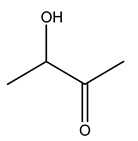	Pruning [16]
2-Butenal-2-ethenyl	C_6_H_8_O	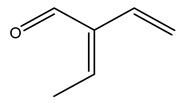	Pruning [13]
2-Butene-1,4-diol	C_4_H_8_O_2_	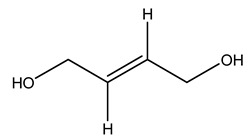	Pruning [16]
Citric acid	C_6_H_8_O_7_	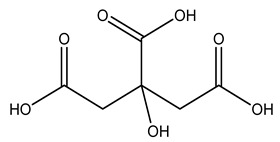	Wood [3] Pruning [21]
1,2-Ethanediol, diacetate	C_6_H_10_O_4_	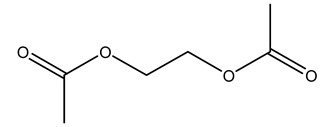	Pruning [16]
Formic acid	CH_2_O_2_	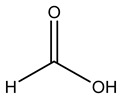	Pruning [16,19,21]
Gluconic acid	C_6_H_12_O_7_	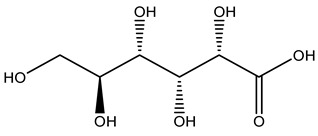	Wood [3]
2,5-Hexanedione	C_6_H_10_O_2_	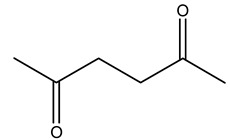	Pruning [15]
2,5-Heptanedione	C_7_H_12_O_2_	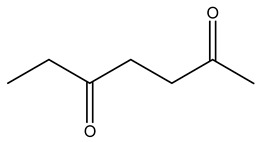	Pruning [12]
3,6-Heptanedione	C_7_H_12_O_2_	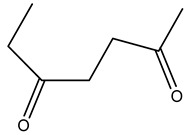	Pruning [12]
Lactic acid	C_3_H_6_O_3_	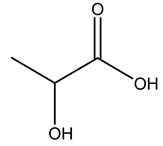	Pruning [21]
Malic acid	C_4_H_6_O_5_	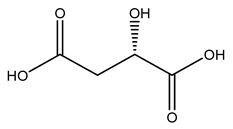	Wood [3]
Methyl acetate	C_3_H_6_O_2_	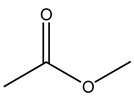	Pruning [16]
Methyl hydroxyacetate	C_3_H_6_O_3_	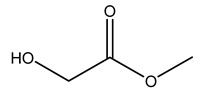	Pruning [16]
3-Octanone	C_8_H_16_O	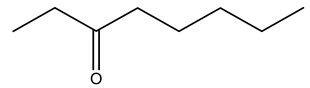	Pruning [12]
2-Oxobutyl acetate	C_6_H_10_O_3_	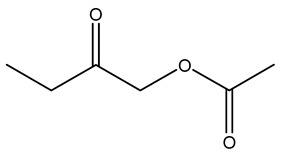	Pruning [16]
Oxopropanoate, ethyl	C_5_H_8_O_3_	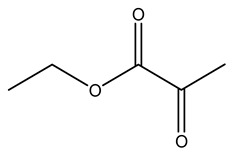	Pruning [16]
Oxopropanoate, methyl	C_4_H_6_O_3_	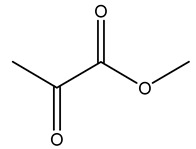	Pruning [16]
2-Oxopropyl acetate	C_5_H_8_O_3_	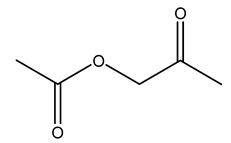	Pruning [16]
Pentanal	C_5_H_10_O	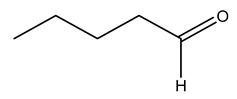	Pruning [16]
2,3-Pentanedione	C_5_H_8_O_2_	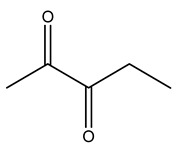	Pruning [16]
Pentanoic acid	C_5_H_10_O_2_	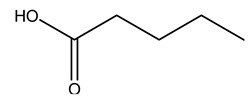	Pruning [12]
Propanoate, vinyl	C_5_H_8_O_2_	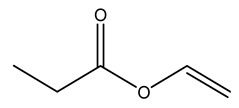	Pruning [16]
2-Propanone, 1-hydroxy	C_3_H_6_O_2_	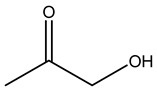	Pruning [16]
2-Propenoic acid	C_3_H_4_O_2_	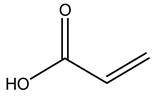	Pruning [16]
Propenoic acid	C_3_H_4_O_2_	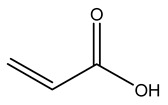	Pruning [16]
1,2-Cyclopentanedione, 3-methyl	C_6_H_8_O_2_	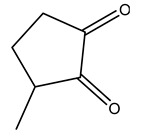	Pruning [16]
Cyclopentanone	C_5_H_8_O	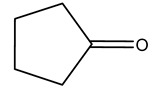	Pruning [16]
2-Cyclopenten-1-one, 3-ethyl-2-hydroxy	C_7_H_10_O_2_	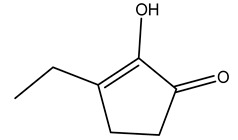	Pruning [16]
2-Cyclopenten-1-one, 2-hydroxy-3-methyl	C_6_H_8_O_2_	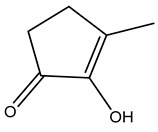	Pruning [13,15,16]
2-Cyclopenten-1-one, 2-methyl	C_6_H_8_O	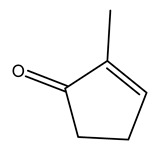	Pruning [13,16]
2-Cyclopentenone, 2-hydroxy-3-ethyl	C_7_H_10_O_2_	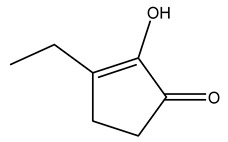	Pruning [12]
2-Cyclohexen-1-one	C_6_H_8_O	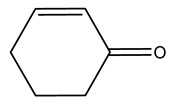	Pruning [16]
Cyclohexanone	C_6_H_10_O	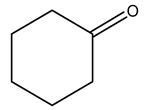	Pruning [16]
Maltol	C_6_H_6_O_3_	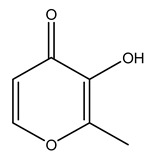	Pruning [15,16]
Styrene	C_8_H_8_	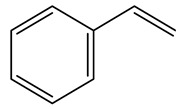	Pruning [16]
Squalene	C_30_H_50_	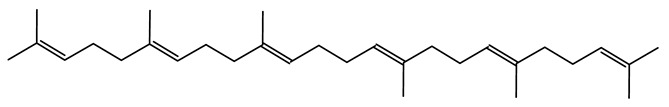	Pruning [13]

**Table 2:** European Standard EN 14961-2 to produce non-industrial pellets.

Parameter	Classification		
Size mm (diameter and length)	A1	A2	B
	D06: D ≤ 6 ± 1 AND 3.15 ≤ L ≤ 40 D08: D ≤ 8 ± 1 AND 3.15 ≤ L ≤ 40		
Moisture content (%)	M10 ≤ 10		
Ash content (%)	A 0.7 ≤ 0.7	A 1.5 ≤ 1.5	A 3.0 ≤ 3.0
N (%)	N 0.3 ≤ 0.3	N 0.5 ≤ 0.5	N 1.0 ≤ 1.0
S (%)	S 0.03 ≤ 0.03	S 0.03 ≤ 0.03	S 0.04≤ 0.04
CI (%)	CI 0.02 ≤ 0.02	CI 0.02 ≤ 0.02	CI 0.03 ≤ 0.03
Durability	DU97.5 ≥ 97.5	DU 97.5 ≥ 97.5	DU 96.5 ≥ 96.5
Bulk density (kg/m^3^)	BD 600 ≥ 600		
Lower heating value	Q 16.5	Q 16.3	Q 16.0
(MJ/kg)	16.5 ≤ Q ≤19	16.3 ≤ Q ≤19	16.0 ≤ Q ≤ 19
Additives	≤2% (type and quantity to be specified)		

**Table 3:** Characteristics of olive pruning (OP) and olive wood (OW) pre-pelletizing.

Characteristics	OP		OW	
Parameters	Avg	SD	Avg	SD
Moisture	10.89	0.16	7.42	0.19
Ash content (%)	5.5	0.06	1.43	0
Volatile content (%)	79.8	0.1	90.15	0
Fixed carbon (%)	3.81	0.2	1	0.19
C	47.12	0.47	46.49	0.11
N	1.11	0.1	0.32	0.01
H	6.82	0.06	6.77	0.04
S	0.05	0	0	0
HHV	18.2	0.01	17.53	0.32
LHV	16.55	0.01	16.06	0.32
Chlorine content as received (%)	0.02	0	0.01	0
Na_2_O	0.135	–	0.092	–
MgO	38.490	–	21.420	–
Al_2_O_3_	0.558	–	0.268	–
SiO_2_	17.200	–	0.813	–
P_2_O_5_	34.600	–	30.000	–
K_2_O	85.390	–	184.400	–
CaO	592.300	–	521.500	–
TiO_2_	0.059	–	0.021	–
Fe_2_O_3_	0.689	–	0.772	–
Initial deformation Temperature (IDT) (°C)	1216	–	1165	–
Spherical temperature (ST) (°C)	1495	–	1493	–
Spherical				
Hemispherical Temperature (HT) (°C)	>1500	–	>1500	–
Fluid temperature (FT) (°C)	>1500	–	>1500	–

**Table 4:** Percentage of pellet samples that satisfy the criteria present in EN 14961-2 for pellet quality.

Parameter	Pellet Sample (%)							
	OP				OW			
	A1	A2	B	NC	A1	A2	B	NC
Durability	42.3		23.1	34.6	91.7		0	8.3
Diameter	100			0	100			0
Length	100			0	100			0
Bulk density	38.5			61.5	83.4			16.6
Moisture content	84.6			15.4	83.4			16.6

**Table 5:** Checklist for assessment of risk of bias in pre-clinical studies [17,126].

Checklist for Assessment of the Risk of Bias in Pre-Clinical Studies
Are the hypothesis and objective of the study clearly described?
Are the main outcomes to be measured clearly described?
Are the main findings of the study clearly described?
Are the samples size calculations reported?
Are the animals randomly housed during the experiment?
Are the investigators blinded from knowledge which treatment used?
Are the outcome assessors blinded?
Is the dose/route of administration of the *O. europea* properly reported?
Is the dose/route of administration of the drug in co-treatment properly reported?
Is the frequency of treatments adequately described?

**Table 6:** Checklist for assessment of the risk of bias in clinical studies [127].

Checklist for Assessment of the Risk of Bias in Clinical Studies
Are the hypothesis and objective of the study clearly described?
Are the main outcomes to be measured clearly described?
Are the main findings of the study clearly described?
Are the samples size calculations reported?
Are the subjects randomly housed during the experiment?
Are the investigators blinded from knowledge which treatment used?
Are the outcome assessors blinded?
Is the dose/route of administration of the *O. europea* properly reported?
Is the dose/route of administration of the drug in co-treatment properly reported?
Is the frequency of treatments adequately described?

**Table 7:** Checklist for assessment of the risk of bias in environmental studies [18,19].

Checklist for Assessment of the Risk of Bias in Environmental Studies
Is the part of the tree clearly described?
Is there a transparent and systematic procedure for the selection of sample plot location (e.g., randomization, grids, etc.,)?
Is the type of study design clearly described (Control-Exposure (CE), Control-Intervention (CI), Before-After-Control-Exposure (BACE), Before-After-Control-Intervention (BACI), Before-After-Intervention (BAI))?
Is the method clearly described?
Is there replication of the measurements?
Are there confounding factors?

## Data Availability

Not applicable.

## Abbreviations

IVDOM	in vitro digestible organic matter
ME	metabolizable energy
NEL	net energy lactation
PEG	polyethylene glycol
WPC	wood-polymer composite
BBA	biomass bottom ash
BFA	biomass fly ash
TCF	totally chlorine free
